# Iterative List Patterned Reed-Muller Projection Detection-Based Packetized Unsourced Massive Random Access

**DOI:** 10.3390/s23146596

**Published:** 2023-07-21

**Authors:** Wenjiao Xie, Runhe Tian, Huisheng Zhang

**Affiliations:** 1 School of Electronics and Information, Northwestern Polytechnical University, Xi’an 710129, China; zhanghuisheng@nwpu.edu.cn; 2 Department of Architecture and Built Environment, University of Nottingham, Nottingham NG7 2RD, UK; runhe.tian@nottingham.ac.uk

**Keywords:** machine-to-machine communications, complex Reed-Muller codes, unsourced massive random access, error correction code

## Abstract

In this paper, we consider a slot-controlled coded compressed sensing protocol for unsourced massive random access (URA) that concatenates a shared patterned Reed–Muller (PRM) inner codebook to an outer error-correction code. Due to the limitations of the geometry-based decoding algorithm in single-sequence settings and due to the message interference that may result in decreased decoding performance under multi-sequence circumstances, a list PRM projection algorithm and an iterative list PRM projection algorithm are proposed to supplant the signal detector associated with the inner PRM sequences in this paper. In detail, we first propose an enhanced path-saving algorithm, called list PRM projection detection, for use in single-user scenarios that maintains multiple candidates during the first few layers so as to remedy the risk of spreading errors. On this basis, we further propose an iterative list PRM projection algorithm for use in multi-user scenarios. The vectors for PRM codes and channel coefficients are jointly detected in an iterative manner, which offers significant improvements regarding the convergence rate for signal recovery. Furthermore, the performances of the proposed algorithms are analyzed mathematically, and we verify that the theoretical simulations are consistent with the numerical simulations. Finally, we concatenate the inner PRM codes that employ iterative list detection in two practical error-correction outer codes. According to the simulation results, we conclude that the packetized URA with the proposed iterative list projection detection works better than benchmarks in terms of the number of active users it can support in each slot and the amount of energy needed per bit to meet an expected error probability.

## 1. Introduction

One of the most prominent uses for 6G wireless networks remains massive machine-type communication’s (mMTC+) continuous evolution [1]. Indeed, mMTC+ services feature the presence of a large number of machines that sporadically link to the system while carrying short data packets. Notably, they are battery-limited and are intended to achieve low transmission latency [2,3,4].

Grant-free transmission [5,6], in which packets are transmitted to the network without informing the base station in advance, is the approach that provides the most promise. “Sourced Random Access” [7] uses grant-free transmission and allocates distinct dictionaries to different users.

Given that there are so many users, employing various encoders would complicate reception since the decoder would first have to determine which encoders were being used. Therefore, using the same coding technique for all users is the most promising strategy. These systems are referred to as “Unsourced Random Access (URA)” [8]. In this way, the identification and decoding responsibilities are decoupled, and the URA receiver only needs to decode the messages without knowing who sent them. A comparison of uncoordinated/unsourced grant-free schemes and coordinated/sourced grant-free (based on compressed sensing technique) schemes in terms of application scenarios, typical access performance, and their characteristics is shown in Table 1. All active users’ per-user probability of error (PUPE) is used to examine the access and transmission performance of the entire system.

The fundamental limits of the Gaussian multiple-access channel are described in Reference [8], which formulates the URA problem from an information-theoretic perspective. Based on Reference [8], an asymptotic improvement is presented in Reference [13]. Both examine a scenario in which the receiver has a fixed count of active users. These findings are expanded in Reference [14] to a case in which the receiver’s active user count is arbitrary and unknowable. For the Gaussian multiple-access channel, a range of low-complexity URA algorithms has been suggested. The main schemes are *T*-fold irregular repetition (which includes the *T*-fold irregular repetition slotted ALOHA protocol with collision detection [15,16]), the sparse interleave division multiple access (IDMA) scheme [17], random spreading and correlation-based energy detection [18,19], as well as the coded compressed sensing (CCS)-based URA scheme [20]. Coded compressed sensing is a divide-and-conquer strategy that concatenates the inner codes to outer A-channel codes, where a tree code is a specific instance of an outer A-channel code devised for this kind of application [21,22]. This makes it simple to accommodate novel channel models owing to the concatenated form’s adaptability to URA. Several later investigations on URA employed an outer A-channel code [23,24,25,26,27,28]. In paper [29], the study scope of a tree code behaving as the outer A-channel code is extended through the integration of the approximate message passing (AMP) mechanism with sparse regression codes [20]. The coded compressed sensing approach is further enhanced in Reference [23] by enabling the inner AMP decoder and the outer tree decoder to communicate soft information through a common message-passing protocol. In order to simplify the high-dimensional sparse signals’ joint detection pertaining to several bases, the research work in Reference [30] suggests a coded demixing method.

Compressed sensing has made extensive use of codebook construction techniques related to second-order Reed–Muller (RM) sequences [31]. RM sequences are excellent options for massive access scenarios during the continuous evolution of massive machine-type communication owing to their high capacity and capability for low-complexity random access [32].

In Reference [33], non-orthogonal Reed–Muller sequences are used as user identifiers (IDs) for active user identification in the grant-based access mechanism in mMTC+, which has low access potency and high signaling expenses. In Reference [34], a strategy for massive random access is proposed that makes use of the nested characteristics of RM codes. Additionally, the coded compressed sensing scheme makes extensive use of inner codebook construction techniques related to second-order RM sequences [35,36], i.e., RM sequences are utilized as inner codes for the CCS protocol, and the outer tree codes are employed for coupling messages for slotted transmissions. In Reference [37], a shared patterned Reed–Muller (PRM) codebook that embeds zero patterns in the second-order RM codes according to a binary vector space partition principle is employed as the inner codebook for the coded compressed sensing protocol.

This paper is inspired by a slot-controlled CCS protocol that concatenates outer error-correction codes to a common PRM inner codebook [37]. In this scheme, a slot-pattern-control (SPC) criterion that corresponds one-to-one to an information segment to construct the slot occupation guideline for each active user. The users’ messages are partitioned into several sub-blocks and add the same SPC segment in each block as a prefix to make up the input signal of the inner encoder. On this basis, each sequence is related to a single codeword from a proposed common codebook called patterned Reed–Muller codes. For recovering the PRM sequences received at the receiver, an algorithm exploiting the geometry of PRM sequences is proposed.  Moreover, the outer tree code is replaced with an error-correcting code, and codewords with a distance at most *t* from the channel output are recovered by outer codes. In view of this scheme, it is anticipated that there is a potential rise in the efficiency of the PRM sequence-related geometry-based decoding algorithm for single-sequence scenarios due to the limited number of active users that can be served over a slot. In addition, message interference may also lead to a decreased decoding efficiency in multi-sequence scenarios. In view of these shortcomings, we replace the inner decoder related to PRM with a proposed list PRM projection algorithm to enhance the efficiency of the inner decoder for a single sequence. Additionally, we construct an iterative list PRM projection algorithm in order to decrease the multi-interference between sequences for the multi-sequence senario. We then deduce the theoretically successful detection probabilities of the proposed algorithm and validate its correctness via numerical simulations. Subsequently, we demonstrate that the proposed schemes can accommodate more active users within each slot using numerical simulations. We finally assess the performance of the slot-controlled URA system with the proposed iterative list algorithm as the inner code detector. According to simulation results, we demonstrate that the schemes using the proposed detection can help improve the overall system’s performance.

### 1.1. Contributions and Arrangement

Here is a summary the contributions of this article:

- A shared PRM codebook that embeds zero patterns in the second-order RM codes according to a binary vector space partition principle is employed as the inner codebook for the coded compressed sensing protocol. On this basis, we propose an enhanced algorithm called the “list PRM projection algorithm” that uses a list of candidates to hinder error spreading in each detection layer.
- As for the multi-sequence scenario, an iterative list PRM projection algorithm is proposed. More specifically, except for the user’s information during the current loop, we consider all signals as interference. We remove the interference in priority and then employ the proposed list projection algorithm to recover the PRM sequence. The PRM estimations are then inserted into the channel estimator to enhance precision. After that, the modified channel estimations are utilized in the subsequent iteration to improve PRM detection further. As a result, the proposed iterative list PRM projection algorithm offers significant advantages regarding the convergence rate.
- The theoretical successful detection probabilities of the proposed list projection and the iterative list projection algorithms are analyzed mathematically, and the results demonstrate that: (i) The factors that affect the efficiency of the list PRM projection algorithm are shown in the simulation results, which validate that the recovery reliability of the first few layers is crucial to the whole performance; (ii) Simulation results further explore the effect of the relationship between rank Υ and *m* for PRM sequence properties on the theoretical successful detection probability; (iii) We verified that the theoretical results are consistent with the simulation results.
- We substitute the inner decoder of the scheme [37] with the proposed iterative list PRM projection algorithm. Simulations of the quasi-static Rayleigh fading multiple-access channel (MAC) are performed numerically, and we conclude the observations as follows: (i) The proposed scheme is superior to the OMP-based inner codes detection, i.e., the chance of successful recovery is significantly improved since the PRM detector and channel estimator share information, and the negative impacts can be further reduced by eliminating those sequences that are incorrectly detected in each operation. (ii) By increasing the count of candidates in the first few layers, we can boost the original PRM projection algorithm’s performance, which further confirms the above proof of the importance of ensuring the reliable recovery of the first few layers.
  (iii) The simulation results of the overall URA system confirm that the packetized URA with the proposed iterative list projection detection method works better than benchmarks in terms of the number of active users it can support in each slot and the amount of energy needed per bit to meet an expected error probability.

The rest of this paper is arranged as follows: In Section 2, we induce the system model and review the patterned Reed–Muller sequences as well as their projection algorithm. An enhanced list PRM projection algorithm and an iterative list PRM projection algorithm are proposed in Section 3. The theoretical probability of the algorithms proposed is derived in Section 4. The simulation results are presented in Section 5, and the paper is concluded in Section 6.

### 1.2. Conventions

It is stipulated that all vectors, whether complex or binary, will be column vectors. We employ $F_{2}^{m}$ to present the finite (binary) field in this paper. $P_{m}$ denotes the size of an m×m matrix.

$P^{T}$ stands for the transpose of matrix P and $P^{-T}$ stands for the inverse transpose of matrix P. The superscript *H* stands for the conjugate transpose of a matrix. Vectors $0_{N}$ and $1_{N}$ are all-zero and all-one vectors of size N×1, respectively. We use $A={\left[a_{i}\right]}_{i=1}^{I}$ to stand for a vector whose capacity is *I*, consisting of the components $a_{i}$(i=1,⋯,I). The vectors $B_{1}\in C^{T_{1}\times 1}$ and $B_{2}\in C^{T_{2}\times 1}$ are added together to produce $B=\left[B_{1};B_{2}\right]\in C^{(T_{1}+T_{2})\times 1}$. $\mathrm{CN}\left(0,I_{N}\right)$ stands for a complex standard normal random vector. The notation $x∼N(\mu ,\sigma^{2})$ indicates that *x* is a Gaussian random variable whose mean is μ and variance is $\sigma^{2}$; its probability density function (PDF) is written as $f_{N}\left(x;\mu ,\sigma^{2}\right)=\frac{1}{\sqrt{2\pi \sigma^{2}}}\exp\left(-\frac{{\left(x-\mu \right)}^{2}}{2\sigma^{2}}\right)$, while $x∼\mathrm{CN}(\mu ,\sigma^{2})$ denotes that the random variable *x* follows the circular symmetric complex Gaussian distribution with its PDF being $f_{CN}\left(x;\mu ,\sigma^{2}\right)=\frac{1}{\pi \sigma^{2}}\exp\left(-\frac{{\left|x-\mu \right|}^{2}}{\sigma^{2}}\right).$

## 2. System Model

In this section, we first review the studies of the slot-controlled URA scheme in Reference [37] and then propose an enhanced slot-controlled URA scheme. In the scheme, each active user partitions the message into several sub-blocks and repeatedly adds a slot-pattern-controlled (SPC) sequence as a prefix to form an outer encoder input signal. Then, error correction encoding is employed to stitch the message through all sub-blocks. A geometry-based technique is employed to detect the sub-blocks transmitted by active users in all sub-slots, and an error correction algorithm stitches these sub-blocks in order to recover the original messages.

### 2.1. Encoding Process

The transmission strategy includes two encoders: an inner-code encoder and an error-correction code/outer-code encoder. The error-correction code encoder uses two practical outer A-channel codes, i.e., the t-tree code and the Reed–-Solomon code with Guruswami–-Sudan list decoding. The inner encoder maps each sub-block into a codeword in the common PRM codebook.

**Figure 1:**
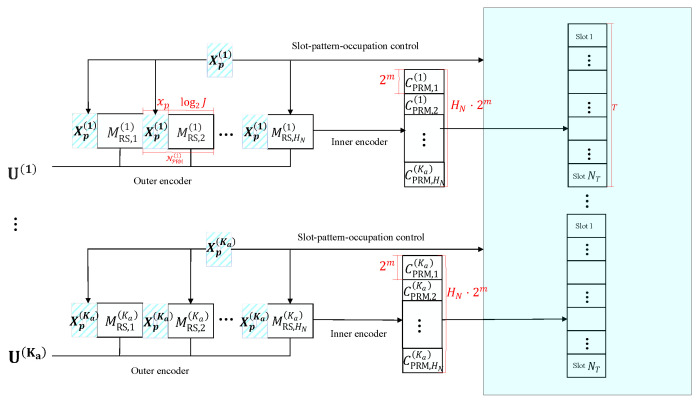
The procedures of the transmitter for the slot-control URA.

As for the transmitter of practical transmission, a *B*-bit message is carried by each active user as part of the set $\left\{W_{1},W_{2},\cdots ,W_{K_{a}}\right\}$ for accessing the system. We let $N=2^{B}$, and each message $W_{k}$ ($1\leq k\leq K_{a}$) is encoded into an $H_{N}$-length outer error-correction code, thus $W_{k}\in \left[N\right]$, where $\left[N\right]=\left\{1,\cdots ,N\right\}$. The A-channel code used for the outer encoding is derived from paper [27]: the *k*th user’s message $U^{\left(k\right)}$ of length *B* is mapped to a *J*-ary Reed–Solomon code of length $H_{N}$, designated ${\left[{\underline{M}}_{\mathrm{RS},h}^{\left(k\right)}\right]}_{h=1}^{H_{N}}=\left(M_{\mathrm{RS},1}^{\left(k\right)},M_{\mathrm{RS},2}^{\left(k\right)},\cdots ,M_{\mathrm{RS},H_{N}}^{\left(k\right)}\right)\in {\left[J\right]}^{H_{N}}$, $0\leq h\leq H_{N}$. We then repeatedly append the binary prefix sequence $X_{p}^{\left(k\right)}$ to each *J*-ary bit $M_{\mathrm{RS},h}^{\left(k\right)}$ to create the *G*-ary sequence. Thus, the length of the binary sequence $X_{p}^{\left(k\right)}$ is set to $\log_{2}\left(G/J\right)$ and we denote $x_{p}=\log_{2}\left(G/J\right)$. For further interpretation, a *G*-ary bit is divided into $2^{x_{p}}$ cosets, each containing $2^{J}$ components, and this process can be summarized as ${\overset{˘}{m}}_{\mathrm{PRM},h}^{\left(k\right)}=𝘍\left(\left[X_{p}^{\left(k\right)};M_{\mathrm{RS},h}^{\left(k\right)}\right]\right)$, where 𝘍(·) is the bijective mapping: $𝘍\left(\cdot \right):\left[2^{x_{p}}\right]\times \left[J\right]$ and ${\overset{˘}{m}}_{\mathrm{PRM},h}^{\left(k\right)}\in G$. By repeating the above processes $H_{N}$ times, an $H_{N}$-length sequence ${\left[{\underline{m}}_{\mathrm{PRM},h}^{\left(k\right)}\right]}_{h=1}^{H_{N}}=\left({\overset{˘}{m}}_{\mathrm{PRM},1}^{\left(k\right)},{\overset{˘}{m}}_{\mathrm{PRM},2}^{\left(k\right)},\cdots ,{\overset{˘}{m}}_{\mathrm{PRM},H_{N}}^{\left(k\right)}\right)$ is obtained, which serves as the input sequence for the inner encoder. We require one-to-one matching between ${\overset{˘}{m}}_{\mathrm{PRM}}^{\left(k\right)}$ and codeword $C_{\mathrm{PRM}}^{\left(k\right)}$ from the common codebook (we omit the subscript *m* here for simplicity), and, herein, ${\overset{˘}{m}}_{\mathrm{PRM}}^{\left(k\right)}$ is the input signal of the inner encoder while the output codeword is $C_{\mathrm{PRM}}^{\left(k\right)}$. Finally, the output of inner coding over *H* slots is recorded as ${\left[{\underline{C}}_{\mathrm{PRM},h}^{\left(k\right)}\right]}_{h=1}^{H_{N}}=\left(C_{\mathrm{PRM},1}^{\left(k\right)},C_{\mathrm{PRM},2}^{\left(k\right)},\cdots ,C_{\mathrm{PRM},H_{N}}^{\left(k\right)}\right)$. This process of the transmitter is depicted in Figure 1. In detail, a length-$N=2^{m}$ sequence $C_{\mathrm{PRM},h}^{\left(k\right)}$ represents an inner PRM code randomly picked from a shared codebook Γ that is sent by the *k*-th user within the *n*-th chunk, where $1\leq n\leq H_{N}$. There is a one-to-one match between the sequence $C_{\mathrm{PRM},h}^{\left(k\right)}$ and a partitioned message of length $B/H_{N}$. Next, using the slot-pattern-control rule, the $H_{N}$ PRM sequences are arranged into $N_{T}$ chunks, which leads to the system employing $T=N\cdot N_{T}$ channel uses to operate a slotted-controlled transmission.

The inner codeword in the *h*-th slot $C_{\mathrm{PRM},h}^{\left(k\right)}$ is derived as follows: a new version of the binary subspace chirp (BSSC) [38,39,40], which embeds zero patterns in the second-order RM codes according to the partition rule of the binary vector space, is proposed by reducing the subspace matrix $H_{\chi }$ to $I_{\chi }$, where χ⊆[m] indicates all possible subsets of rank Υ∈{1,2,⋯,m} under full dimension *m*. The simplified sequence is denoted as patterned Reed–Muller (PRM) and can be written as follows

(1) $$C_{\mathrm{PRM},h}^{\left(k\right)}\left(v\right)=\left\{\begin{array}{cc} \frac{1}{\sqrt{2^{\Upsilon }}}i^{2w^{T}{B|}_{1}^{\Upsilon }+w^{T}\hat{P}w},\; & \;\mathrm{if}\;v=I_{\chi }w+\tilde{I_{\chi }}{B|}_{\Upsilon +1}^{m},\\ 0,\; & \;\mathrm{otherwise}\;, \end{array}\right.$$

where $w\in F_{2}^{\Upsilon }$, symmetric matrix $\hat{P}$ and vector B are dominant parameters for RM (Υ,2); additionally, sub-vector ${B|}_{\Upsilon +1}^{m}$ identifies the sequence of the last (m−Υ)-bit segment of the *m*-length vector B, and subspace $I_{\chi }$ is a matrix of size (m×Υ) with column vectors $e_{\chi (1)},e_{\chi (2)},\cdots ,e_{\chi (\Upsilon )}$, where $e_{\chi (r)}$ is the unit vector with non-zero at the *r*-th position, 1≤r≤Υ. Similarly, matrix $\tilde{I_{\chi }}$ follows the same rule.

### 2.2. Decoding Process

The signal received in the receiver for the *n*-th slot ($1\leq n\leq N_{T}$) is expressed as

(2) $$y_{n}=\sum_{k=1}^{K_{a}}h_{k,n}\cdot x_{k,n}+n_{n},$$

where $h_{k,n}∼CN\left(0,1\right)$ represents a constant channel coefficient that is supposed to be stable for the duration of a user’s transmission between the active user *k* and the access point (AP) in *n*-th chunk. While, $n_{n}∼CN\left(0,I_{N}\right)$ is the complex additive white Gaussian noise (AWGN). For the inner-code decoder: PRM codes have their geometry property: for any $z\in F_{2}^{m-\Upsilon }$, the PRM sequence satisfies

(3) $$E\left(I_{\chi }e_{r},\tilde{I_{\chi }}z+I_{\chi }{\hat{P}}_{r}\right)\cdot C_{\hat{P},B,I_{\chi }}={\left(-1\right)}^{B^{T}\left(e_{r};0^{m-\Upsilon }\right)+{\left(B{|}_{\Upsilon +1}^{m}\right)}^{T}z}\cdot C_{\hat{P},B,I_{\chi }},$$

where ${\hat{P}}_{r}=\hat{P}e_{r}$. According to Equation (3), the PRM sequence obtains its specific projective property by letting $z=0^{m-\Upsilon }$:

(4) $$\lambda_{PRM,\varpi }^{\left(r\right)}\left(0^{m-\Upsilon }\right)\cdot C_{\hat{P},B,I_{\chi }}=\left\{\begin{array}{cc} C_{\hat{P},B,I_{\chi }},\; & \;\mathrm{if}\;\varpi ={\left(-1\right)}^{B_{r}},\\ 0,\; & \;\mathrm{if}\;\varpi \neq {\left(-1\right)}^{B_{r}}, \end{array}\right.$$

where

(5) $$\lambda_{PRM,\varpi }^{\left(r\right)}\left(0^{m-\Upsilon }\right)=\frac{\left[I_{N}+\varpi \cdot E\left(I_{\chi }e_{r},I_{\chi }{\hat{P}}_{r}\right)\right]}{2}$$

is a projection operator for PRM sequences. We decode the inner code using the OMP and the projective-based algorithm. Owing to the slot-controlled URA, the inner-code sequence based on projective property and the channel estimations are recovered sequentially. For the outer-decoding process, the number of output codewords is equivalent to the count of decoding iterations $K_{0}$. The outer Reed–Solomon decoding process is suggested. The efficiency of the method described above can be improved using a modified version based on list decoding, which will be described in the next part.

We assess performance from the perspective of messages and use the miss-detection rate (MDR) and the false-alarm rate (FAR) as the primary indicators, which are given as

(6) $$P_{e}≜\frac{1}{K_{a}}\sum_{k\in K_{a}}\mathrm{Pr}\left[W_{k}\notin \hat{D}\left(Y\right)\right]$$

and

(7) $$P_{f}≜\mathrm{Pr}\left[\hat{D}\left(Y\right)\left\{W_{k}|k\in K_{a}\right\}\neq \emptyset \right],$$

respectively, where $K_{a}=\left\{1,\cdots ,K_{a}\right\}$.

## 3. Iterative List PRM Projection Algorithm-Based Inner-Code Detector

Our proposed detection scheme for inner codes is described in this section. In addition to the modified list PRM projection detection adopted for a single sequence, we propose an iterative list PRM projection algorithm implemented at the AP for multiple sequences.

### 3.1. List PRM Projection Algorithm

The received signal is $y=C_{\hat{P},B,I_{\chi }}+n$, and the enhanced algorithm based on the detection proposed in Reference [37] is executed for the single-user scenario. Specifically, in this single-user scenario, we propose a modified algorithm that utilizes a candidate-saving technique during each layer of detection to boost performance. Algorithm 1’s particular processes are outlined in the following section.

Three elements $\left\{I_{\chi },\hat{P},B\right\}$ of the PRM sequence need to be recovered, and the subspace matrix $I_{\chi }$ should be retrieved first. Since the rank is unknown, we go over every conceivable rank in 1≤Υ≤m and set aside *L* potential subspace matrices for each one (in Lines 3 and 4) after we calculate $F_{\mathrm{sum}}\left(f_{\chi_{\left(\Upsilon \right)}}\right)=\sum_{j=1}^{2^{\left(m-\Upsilon \right)}}\left|y^{H}E\left(0,f_{\chi_{\left(\Upsilon \right)}}^{\left(j\right)}\right)y\right|$ for every $f_{\chi_{\left(\Upsilon \right)}}^{\left(j\right)}\in F_{2}^{m}$, employ the relation of ${\left[f_{\chi_{\left(\Upsilon \right)}}^{\left(j\right)}\right]}_{j=1}^{2^{\left(m-\Upsilon \right)}}=\mathrm{span}\left({\tilde{I}}_{\chi_{\left(\Upsilon \right)}}\right)$ and seek the biggest $2^{\left(m-\Upsilon \right)}$ estimations of $F_{\mathrm{sum}}$, the set ${\left[f_{\chi_{\left(\Upsilon \right)}}^{\left(j\right)}\right]}_{j=1}^{2^{\left(m-\Upsilon \right)}}$ and the subspace matrix ${\tilde{I}}_{\chi_{\left(\Upsilon \right)}}$ can be identified. We finally keep *L* candidates as $I_{\chi_{\left(\Upsilon \right)}}=\left\{{\left[{\tilde{I}}_{\chi_{\left(\Upsilon \right)}}^{\left(l\right)}\right]}_{l=1}^{L},\Upsilon \right\}$.

The recovery of remaining elements $\left\{\hat{P},B\right\}$ depends on the subspaces in $I_{\chi_{\left(\Upsilon \right)}}$. In the following, we will decode the vector $\hat{P_{l}}$ and bit $B_{l}$ in a layer-by-layer manner. Furthermore, we will keep *H* parent candidates for the next layer at the end of each iteration. We initialize the corresponding parameters in Line 6; we denote the list of updated symmetric matrix and vector (under ${\tilde{I}}_{\chi_{\left(\Upsilon \right)}}^{\left(l\right)}$) as $H_{l}$ during each layer, and update the list of search space as ${\left[F_{l,1}^{\left(h\right)}\right]}_{h=1}^{H}$ using (6). During the iteration, as for each candidate derived from the previous layers, we obtain a list of *H* candidates for the current layer (in Lines 8–11); we first update the search space from the previous knowledge using (6) (in Lines 6 and 19). The detected layers notated as R (R=1,⋯,r−1) and utilize the recovered vector ${\hat{P}}_{r,l}^{\left(h\right)}$ (${\hat{P}}_{r,l}^{\left(h\right)}$ is *h*-th candidates for r-th column vector of $\hat{P}$ under subspace $I_{\chi \left(\Upsilon \right)}^{\left(l\right)}$) to fill out the symmetric matrix; thus, the matrix under the r-th layer can be represented as ${\underline{\hat{P}}}_{R}^{\left(h\right)}$. On this basis, the search space is limited to

(8) $$F_{l,r}^{\left(h\right)}=\left\{f\in F_{2}^{m}|f_{i}={\underline{\hat{P}}}_{R}^{\left(h\right)}\left(i,r\right)\;\mathrm{for}\;\mathrm{all}\;i\in R\right\},$$

where the value of ${\underline{\hat{P}}}_{R}^{\left(h\right)}\left(i,r\right)$ refers to the *i*-th row and r-th column of matrix ${\underline{\hat{P}}}_{R}^{\left(h\right)}$. Next, we derive *H* paths for each candidates of previous decoding (in Lines 9 and 10).

For all vectors $f\in F_{l,r}^{\left(h\right)}$, we compute $F_{\mathrm{sum}}\left(f\right)=\sum_{j=1}^{2^{\left(m-\Upsilon \right)}}\left|y^{H}E\left(I_{\chi_{\left(\Upsilon \right)}}^{\left(l\right)}\cdot e_{r},f\right)y\right|$ to search *H* largest $F_{\mathrm{sum}}$, the corresponding *H* sets of ${\left[f_{j}\right]}_{j=1}^{2^{\left(m-\Upsilon \right)}}$ are derived. Based on ${\left[f_{j}\right]}_{j=1}^{2^{\left(m-\Upsilon \right)}}=\mathrm{span}\left({\tilde{I}}_{\chi_{\left(\Upsilon \right)}}^{\left(l\right)}\right)+I_{\chi_{\left(\Upsilon \right)}}^{\left(l\right)}{\hat{P}}_{r,l}e_{r}$, vector $P_{r,l}^{\left(h\right)}$ can be obtained. Once $2^{H}$ iterations are complete, we finally get the ${\left[{\hat{P}}_{r,l}^{\left(h\right)}\right]}_{h=1}^{H}$ within $2^{H}$ possibles.
**Algorithm 1:** List PRM projection algorithm
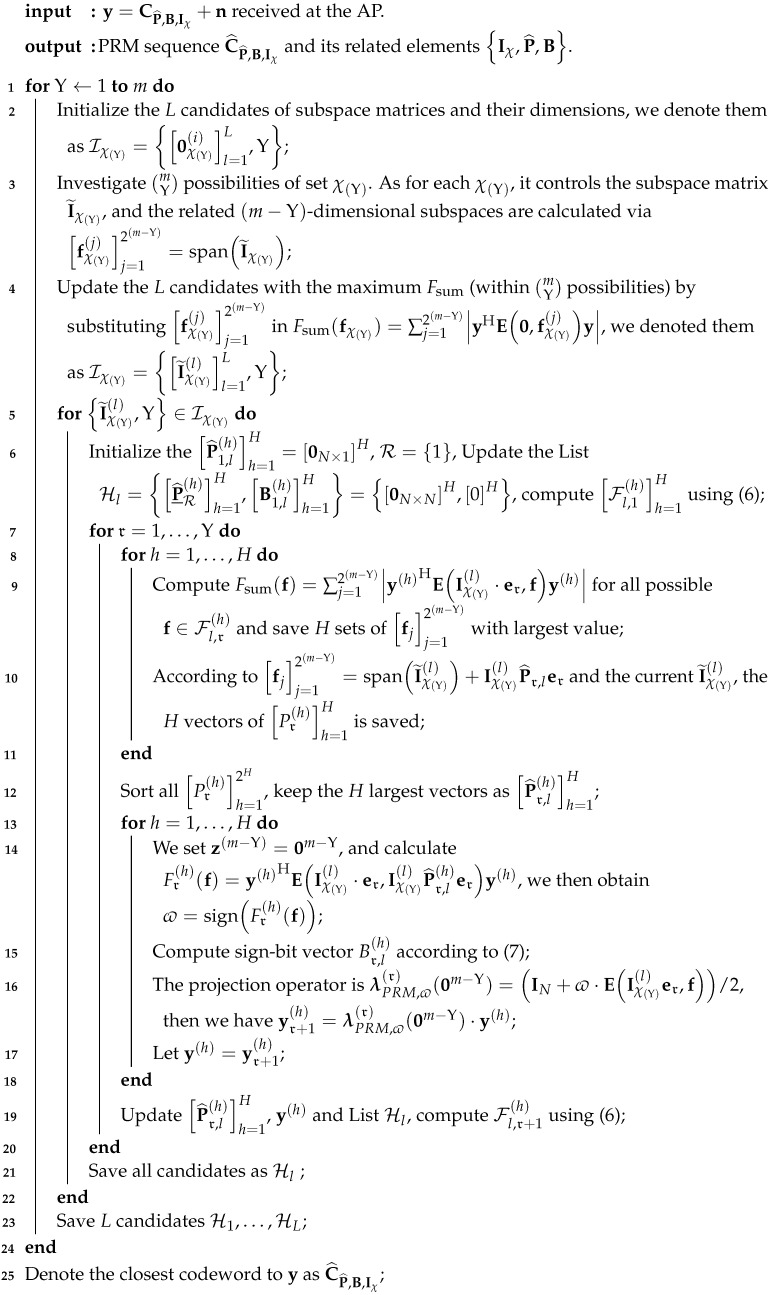


As for the detection of $B_{l}^{\left(h\right)}$ (in Lines 14–17), we make $z^{\left(m-\Upsilon \right)}=0^{m-\Upsilon }$ and compute $F_{r}\left(f\right)=y^{H}E\left(I_{\chi_{\left(\Upsilon \right)}}^{\left(l\right)}\cdot e_{r},I_{\chi_{\left(\Upsilon \right)}}^{\left(l\right)}{\hat{P}}_{r,l}e_{r}\right)y$, then we obtain $\mathrm{sign}\left(F_{r}\left(f\right)\right)={\left(-1\right)}^{B_{r,l}}$. In light of this, $B_{r}$ can be obtained as follows

(9) $$B_{r,l}=\left\{\begin{array}{cc} 1, & \mathrm{if}\;\mathrm{sign}\left(F_{r}\left(f\right)\right)=-1,\\ 0, & \mathrm{if}\;\mathrm{sign}\left(F_{r}\left(f\right)\right)=1. \end{array}\right.$$

Line 19 updates all parameters for the next layer detection. In Line 23, after conducting all iterations, we evaluate each rank’s (L×H) results and retain the optimal results. The final results are selected at the end of the procedure for all ranks (in Line 25).

### 3.2. Iterative List PRM Projection Algorithm

We consider a scenario where the simultaneous presence of multiple active users in a system. As described below, we will detect multiple PRM sequences iteratively. We begin by introducing some notations in this section: it is possible to detect a maximum of $K_{0}$ users. We denote the notation ${\hat{C}}_{\hat{P},B,I_{\chi }}^{\left(s,k\right)}$ as the detected PRM sequence, and ${\hat{h}}_{k}^{\left(s\right)}$ represents the channel estimation. Both notations indicate the case under the *k*-th user and during the *i*-th iteration ($1\leq s\leq S_{max}$). The particular processes of Algorithm 2 are outlined in the  following section.
**Algorithm 2:** Iterative List PRM projection Algorithm
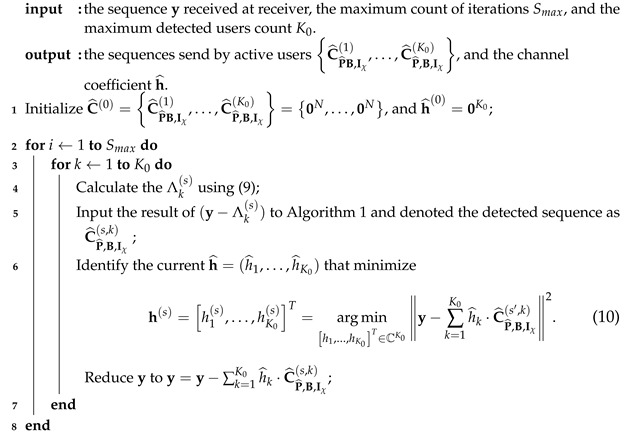


First, the PRM sequence and the channel coefficient are initialized as ${\hat{C}}_{\hat{P},B,I_{\chi }}^{\left(0,k\right)}=0^{N}$ and ${\hat{h}}_{k}^{\left(0\right)}=0$ for s=0, respectively, (in Line 1). We then denote an interference for the user *k* during the *s*-th iteration as

(11) $$\Lambda_{k}^{\left(s\right)}=\sum_{k^{\prime }=1andk^{\prime }\neq k}^{K_{0}}{\hat{h}}_{k^{\prime }}^{\left(s^{\prime }\right)}\cdot {\hat{C}}_{\hat{P},B,I_{\chi }}^{\left(s^{\prime },k^{\prime }\right)},$$

where

(12) $$\left\{\begin{array}{cc} s^{\prime }=s,\; & \;\mathrm{if}\;1\leq k^{\prime }\leq k-1,\\ s^{\prime }=s-1,\; & \;\mathrm{if}\;k+1\leq k^{\prime }\leq K_{0}. \end{array}\right.$$

Then, in order to retrieve a PRM sequence ${\hat{C}}_{\hat{P},B,I_{\chi }}^{\left(s,k\right)}$, we feed the signal ($y-\Lambda_{k}^{\left(s\right)}$) into Algorithm 1.

Next, when detecting each user’s PRM sequence, the detected sequences are inserted into the linear least square channel estimator in order to improve their accuracy further, specified as (8) in Algorithm 2. Due to the exchange of information between the sequence detector and the channel estimator, the effectiveness gradually improves, and it terminates once the results have converged or the maximum number of iterations has been reached.

## 4. Performance Analysis

### 4.1. Successful Recovery Probability of the List PRM Projection Algorithm

For detecting a single PRM sequence, Algorithm 1 recovers the column vectors of the matrix $\hat{P}$ layer by layer. In this section, we will derive the probability of successful recovery of each layer element ${\hat{P}}_{r}$ for 1≤r≤m. The corresponding conclusions are summarized in Lemma 1 as well as Theorem 1.

**Lemma 1.**  *Suppose that Algorithm 1’s input signal is $y=hC_{\hat{P},B,I_{\chi }}+n$, where h∼CN(0,1) is the channel coefficient, and $n∼CN(0,I_{N})$ denotes the complex additive white Gaussian noise (AWGN). The notation $P^{\left(r\right)}\left(1\leq r\leq m\right)$ represents the event “the vector ${\hat{P}}_{r}$ is recovered successful” and the notation $P^{\left(1,\cdots ,r-1\right)}$ denotes the event ” the vectors ${\hat{P}}_{1},\cdots ,{\hat{P}}_{r-1}$ are correctly recovered”. On this basis, if h and $P^{\left(1,\cdots ,r-1\right)}$ is given, then for any $z\in F_{2}^{m-\Upsilon }$, $\mathrm{Pr}\left(P^{\left(r\right)}|P^{\left(1,\cdots ,r-1\right)},h\right)$ can be expressed as*

(13) $$\begin{array}{c} \begin{array}{cc} \; & \mathrm{Pr}\left(P^{\left(r\right)}|P^{\left(1,\cdots ,r-1\right)},h\right)\\ \geq & {\left[Q\left(-\frac{2^{\Upsilon }{\left|h\right|}^{2}\cdot {\left(-1\right)}^{B_{r}+{\left(B{|}_{\Upsilon +1}^{m}\right)}^{T}z}}{\sqrt{2}\sigma_{F,r}}\right)\cdot Q\left(-\frac{2^{\Upsilon }{\left|h\right|}^{2}\cdot {\left(-1\right)}^{B_{r}+{\left(B{|}_{\Upsilon +1}^{m}\right)}^{T}z}}{2\sigma_{F,r}}\right)\right]}^{2^{\left(\Upsilon -r+1\right)}-1}. \end{array} \end{array}$$

*In (13), function Q(·) is $Q\left(x\right)=\int_{x}^{\infty }\frac{1}{\sqrt{2\pi }}\exp\left(-\frac{t^{2}}{2}\right)dt$, and the variance $\sigma_{F,r}^{2}$ is defined as*

(14) $$\sigma_{F,r}^{2}=2^{m-1}\left[{2|h|}^{2}\frac{N_{0}}{2^{r-1}}+{\left(\frac{N_{0}}{2^{r-1}}\right)}^{2}\right].$$

**Theorem 1.**  *Taking into account the channel coefficients h and the results of (13), the average probability of successful detection for Algorithm 1 can be calculated as*

(15) $$P_{succ}=\int_{h}f_{\mathrm{CN}}\left(h;0,1\right)\cdot \overset{ˇ}{P}\left(\Upsilon ,N_{0}|h\right)dh,$$

*where $\overset{ˇ}{P}\left(\Upsilon ,N_{0}|h\right)=\prod_{r=1}^{\Upsilon }\mathrm{Pr}\left(P^{\left(r\right)}|P^{\left(1,\cdots ,r-1\right)},h\right)$ and $f_{\mathrm{CN}}\left(h;0,1\right)=\frac{1}{\pi }\exp\left(-{\left|\mu \right|}^{2}\right)$.*

**Proof**  We assume that the subspace matrix $I_{\chi }$ is decoded correctly and H=1. Define a concept $F_{S}\left(f_{r}\right)$ related to $f_{r}\in F_{2}^{m}$ as

(16) $$\begin{array}{c} \begin{array}{cc} F_{S}\left(f_{r}\right) & ={\left(hC_{\hat{P},B,I_{\chi }}+n\right)}^{H}\cdot E\left(I_{\chi }e_{r},f_{r}\right)\cdot \left(hC_{\hat{P},B,I_{\chi }}+n\right)\\ \; & =\underset{F_{A}\left(f_{r}\right)}{\underset{︸}{{\left|h\right|}^{2}C_{\hat{P},B,I_{\chi }}^{H}E\left(I_{\chi }e_{r},f_{r}\right)C_{\hat{P},B,I_{\chi }}}}+\underset{Z_{1}\left(f_{r}\right)}{\underset{︸}{n^{H}E\left(I_{\chi }e_{r},f_{r}\right)n}}+\underset{Z_{2}\left(f_{r}\right)}{\underset{︸}{h\left[C_{\hat{P},B,I_{\chi }}^{H}E\left(I_{\chi }e_{r},f_{r}\right)n+n^{H}E\left(I_{\chi }e_{r},f_{r}\right)C_{\hat{P},B,I_{\chi }}\right]}}. \end{array} \end{array}$$

According to (16), $Z_{1}\left(f_{r}\right)$ and $Z_{2}\left(f_{r}\right)$ are the sources of interference, and we use $Z\left(f_{r}\right)=Z_{1}\left(f_{r}\right)+Z_{2}\left(f_{r}\right)$ to conclude all interferences. The following discussion will demonstrate Lemma 1 through mathematical induction.

(1) When r=1: according to Algorithm 1, the event $P^{\left(1\right)}$ occurring is described as $\sum_{z\in F_{2}^{m-\Upsilon }}\left|F\left(\tilde{I_{\chi }}z+{\tilde{p}}_{\chi ,1}\right)\right|>\sum_{z\in F_{2}^{m-\Upsilon }}\left|F\left(\tilde{I_{\chi }}z+{\bar{\bar{p}}}_{\chi ,1}\right)\right|$ (we omit the subscript S and record it as F(·) in this section), where ${\tilde{p}}_{\chi ,1}=I_{\chi }\hat{P}e_{1}$, and symbol ${\bar{\bar{p}}}_{\chi ,1}$ denotes any possible vector that is different from ${\tilde{p}}_{\chi ,1}$. It is known that the vector ${\bar{\bar{p}}}_{\chi ,1}$ has a total of $\left(2^{\Upsilon }-1\right)$ possibilities, then we denote the probability of the event $P^{\left(1\right)}$ as follows:

(17) $$\begin{array}{c} \begin{array}{cc} \mathrm{Pr}\left(P^{\left(1\right)}|h\right) & =\mathrm{Pr}\left(\sum_{z\in F_{2}^{m-\Upsilon }}\left|F\left(\tilde{I_{\chi }}z+{\tilde{p}}_{\chi ,1}\right)\right|>\max\sum_{z\in F_{2}^{m-\Upsilon }}\left|F\left(\tilde{I_{\chi }}z+{\bar{\bar{p}}}_{\chi ,1}\right)\right|\right)\\ \; & =\prod_{{\left[{\bar{\bar{p}}}_{\chi ,1}^{\left(j\right)}\right]}_{j=1}^{2^{\Upsilon -1}}}\mathrm{Pr}\left(\sum_{z\in F_{2}^{m-\Upsilon }}\left|F\left(\tilde{I_{\chi }}z+{\tilde{p}}_{\chi ,1}\right)\right|>\sum_{z\in F_{2}^{m-\Upsilon }}\left|F\left(\tilde{I_{\chi }}z+{\bar{\bar{p}}}_{\chi ,1}^{\left(j\right)}\right)\right|\right). \end{array} \end{array}$$

In (17), ${\left[{\bar{\bar{p}}}_{\chi ,1}^{\left(j\right)}\right]}_{j=1}^{2^{\Upsilon -1}}$ is the set of all possible vectors except ${\tilde{p}}_{\chi ,1}$. Since each term within the summation function obeys the same distribution, calculating (17) is equivalent to computing its approximate probability as

(18) $$\begin{array}{c} \begin{array}{c} \prod_{{\left[{\bar{\bar{p}}}_{\chi ,1}^{\left(j\right)}\right]}_{j=1}^{2^{\Upsilon -1}}}\mathrm{Pr}\left(\left|F\left(\tilde{I_{\chi }}z+{\tilde{p}}_{\chi ,1}\right)\right|>\left|F\left(\tilde{I_{\chi }}z+{\bar{\bar{p}}}_{\chi ,1}^{\left(j\right)}\right)\right|\right). \end{array} \end{array}$$

For convenience, we let the notation ${\tilde{\Xi }}_{\chi ,z}^{\left(1\right)}=\tilde{I_{\chi }}z+{\tilde{p}}_{\chi ,1}$ and ${\bar{\bar{\Xi }}}_{\chi ,z}^{\left(1\right)}=\tilde{I_{\chi }}z+{\bar{\bar{p}}}_{\chi ,1}$. Since the $\left(2^{\Upsilon }-1\right)$ events in (18) are independent of one another, we calculate the probability of one event first:

(19) $$\begin{array}{c} \begin{array}{cc} \; & \mathrm{Pr}\left(\left|F\left(\tilde{I_{\chi }}z+{\tilde{p}}_{\chi ,1}\right)\right|>\left|F\left(\tilde{I_{\chi }}z+{\bar{\bar{p}}}_{\chi ,1}\right)\right|\right)\\ \overset{\left(a\right)}{=} & \mathrm{Pr}\left(\left|{\left|h\right|}^{2}C_{\hat{P},B,I_{\chi }}^{H}E\left(I_{\chi }e_{1},{\tilde{\Xi }}_{\chi ,z}^{\left(1\right)}\right)C_{\hat{P},B,I_{\chi }}+Z\left({\tilde{\Xi }}_{\chi ,z}^{\left(1\right)}\right)\right|>\left|Z({\bar{\bar{\Xi }}}_{\chi ,z}^{\left(1\right)})\right|\right)\\ \overset{\left(b\right)}{=} & \mathrm{Pr}\left({\left|h\right|}^{2}C_{\hat{P},B,I_{\chi }}^{H}E\left(I_{\chi }e_{1},{\tilde{\Xi }}_{\chi ,z}^{\left(1\right)}\right)C_{\hat{P},B,I_{\chi }}+Z\left({\tilde{\Xi }}_{\chi ,z}^{\left(1\right)}\right)>\left|Z({\bar{\bar{\Xi }}}_{\chi ,z}^{\left(1\right)})\right|\right)\\ & \cdot \mathrm{Pr}\left({\left|h\right|}^{2}C_{\hat{P},B,I_{\chi }}^{H}E\left(I_{\chi }e_{1},{\tilde{\Xi }}_{\chi ,z}^{\left(1\right)}\right)C_{\hat{P},B,I_{\chi }}+Z\left({\tilde{\Xi }}_{\chi ,z}^{\left(1\right)}\right)>0\right)\\ \; & +\mathrm{Pr}\left(-{\left|h\right|}^{2}C_{\hat{P},B,I_{\chi }}^{H}E\left(I_{\chi }e_{1},{\tilde{\Xi }}_{\chi ,z}^{\left(1\right)}\right)C_{\hat{P},B,I_{\chi }}-Z\left({\tilde{\Xi }}_{\chi ,z}^{\left(1\right)}\right)>\left|Z({\bar{\bar{\Xi }}}_{\chi ,z}^{\left(1\right)})\right|\right)\\ & \cdot \mathrm{Pr}\left({\left|h\right|}^{2}C_{\hat{P},B,I_{\chi }}^{H}E\left(I_{\chi }e_{1},{\tilde{\Xi }}_{\chi ,z}^{\left(1\right)}\right)C_{\hat{P},B,I_{\chi }}+Z\left({\tilde{\Xi }}_{\chi ,z}^{\left(1\right)}\right)<0\right)\\ \overset{\left(c\right)}{\geq } & \mathrm{Pr}\left({\left|h\right|}^{2}C_{\hat{P},B,I_{\chi }}^{H}E\left(I_{\chi }e_{1},{\tilde{\Xi }}_{\chi ,z}^{\left(1\right)}\right)C_{\hat{P},B,I_{\chi }}+Z\left({\tilde{\Xi }}_{\chi ,z}^{\left(1\right)}\right)>\left|Z({\bar{\bar{\Xi }}}_{\chi ,z}^{\left(1\right)})\right|\right)\\ & \cdot \mathrm{Pr}\left({\left|h\right|}^{2}C_{\hat{P},B,I_{\chi }}^{H}E\left(I_{\chi }e_{1},{\tilde{\Xi }}_{\chi ,z}^{\left(1\right)}\right)C_{\hat{P},B,I_{\chi }}+Z\left({\tilde{\Xi }}_{\chi ,z}^{\left(1\right)}\right)>0\right), \end{array} \end{array}$$

where the equal sign (a) is based on $C_{\hat{P},B,I_{\chi }}^{H}E\left(I_{\chi }e_{1},{\bar{\bar{\Xi }}}_{\chi ,z}^{\left(1\right)}\right)C_{\hat{P},B,I_{\chi }}=0$, (b) and (c) are obtained according to the following equations:

(20) $$\begin{array}{c} \begin{array}{cc} \mathrm{Pr}(|A|\geq |B|)= & \mathrm{Pr}(A>|B|)\mathrm{Pr}(A>0)+\mathrm{Pr}(-A>|B|)\mathrm{Pr}(A<0)\\ \geq & \mathrm{Pr}(A>|B|)\mathrm{Pr}(A>0), \end{array} \end{array}$$

and ||A|−|B||≤|A±B|≤|A|+|B|.

In order to calculate the two probabilities of the result of (19), it is necessary to study the distribution of the variables ${\left|h\right|}^{2}C_{\hat{P},B,I_{\chi }}^{H}E\left(I_{\chi }e_{1},{\tilde{\Xi }}_{\chi ,z}^{\left(1\right)}\right)C_{\hat{P},B,I_{\chi }}$, $Z({\tilde{\Xi }}_{\chi ,z}^{\left(1\right)})$ and $Z({\bar{\bar{\Xi }}}_{\chi ,z}^{\left(1\right)})$, respectively. We prioritize the study of ${\left|h\right|}^{2}C_{\hat{P},B,I_{\chi }}^{H}E\left(I_{\chi }e_{1},{\tilde{\Xi }}_{\chi ,z}^{\left(1\right)}\right)C_{\hat{P},B,I_{\chi }}$: we omit the factor $1/2^{r}$ for the representation of $C_{\hat{P},B,I_{\chi }}$ and denote $F_{A}\left({\tilde{\Xi }}_{\chi ,z}^{\left(1\right)}\right)$ as follows

(21) $$\begin{array}{c} \begin{array}{cc} F_{A}\left({\tilde{\Xi }}_{\chi ,z}^{\left(1\right)}\right) & ={\left|h\right|}^{2}C_{\hat{P},B,I_{\chi }}^{H}E\left(I_{\chi }e_{1},{\tilde{\Xi }}_{\chi ,z}^{\left(1\right)}\right)C_{\hat{P},B,I_{\chi }}\\ \; & ={\left|h\right|}^{2}\cdot i^{{\left(I_{\chi }e_{1}\right)}^{T}\cdot {\tilde{\Xi }}_{\chi ,z}^{\left(1\right)}}\sum_{w\in F_{2}^{m}}\bar{C_{\hat{P},B,I_{\chi }}\left(w+I_{\chi }e_{1}\right)}\cdot {\left(-1\right)}^{w^{T}\cdot {\tilde{\Xi }}_{\chi ,z}^{\left(1\right)}}\cdot C_{\hat{P},B,I_{\chi }}\left(w\right)\\ & ={\left|h\right|}^{2}\cdot \sum_{w\in F_{2}^{m}}i^{2{\left({\hat{P}}_{1};0\right)}^{T}\cdot Q^{-1}w+P_{1,1}+2B^{T}\left(e_{1};0\right)}\cdot i^{{\left(I_{\chi }e_{1}\right)}^{T}\cdot {\tilde{\Xi }}_{\chi ,z}^{\left(1\right)}}\cdot {\left(-1\right)}^{w^{T}\cdot {\tilde{\Xi }}_{\chi ,z}^{\left(1\right)}}\\ & \cdot \phi \left[B,Q^{-1}w+\left(e_{1};0\right),\Upsilon \right]\cdot \phi \left[B,Q^{-1}w,\Upsilon \right]\\ & ={\left|h\right|}^{2}\cdot i^{2B^{T}\left(e_{1};0\right)+P_{1,1}}\sum_{w\in F_{2}^{m}}i^{2{\left({\hat{P}}_{1};0\right)}^{T}\cdot Q^{-1}w+2w^{T}\cdot {\tilde{\Xi }}_{\chi ,z}^{\left(1\right)}+{\left(I_{\chi }e_{1}\right)}^{T}\cdot {\tilde{\Xi }}_{\chi ,z}^{\left(1\right)}}\\ & \cdot \phi \left[B,Q^{-1}w+\left(e_{1};0\right),\Upsilon \right]\cdot \phi \left[B,Q^{-1}w,\Upsilon \right], \end{array} \end{array}$$

where $P_{1,1}=e_{1}^{T}Pe_{1}=e_{1}^{T}P_{1}$ and $\phi \left(B,Q^{-1}w,\Upsilon \right)=1$ in the case of ${B|}_{\Upsilon +1}^{m}=\left(Q^{-1}w\right){|}_{\Upsilon +1}^{m}$. We then substitute ${\tilde{\Xi }}_{\chi ,z}^{\left(1\right)}=\tilde{I_{\chi }}z+{\tilde{p}}_{\chi ,1}$ in (21), and the equation expression becomes:

(22) $$\begin{array}{c} \begin{array}{cc} F_{A}\left({\tilde{\Xi }}_{\chi ,z}^{\left(1\right)}\right)= & F_{A}\left(\tilde{I_{\chi }}z+{\tilde{p}}_{\chi ,1}\right)\\ = & {\left|h\right|}^{2}\cdot i^{2B^{T}\left(e_{1};0\right)+2P_{1,1}}\cdot \sum_{w\in F_{2}^{m}}i^{2{\left(\tilde{I_{\chi }}z\right)}^{T}w}\cdot \phi \left[B,Q^{-1}w+\left(e_{1};0\right),\Upsilon \right]\cdot \phi \left[B,Q^{-1}w,\Upsilon \right]\\ = & {\left|h\right|}^{2}\cdot i^{2B^{T}\left(e_{1};0\right)+2P_{1,1}}\cdot \sum_{w\in F_{2}^{m}}i^{2{\left(B{|}_{\Upsilon +1}^{m}\right)}^{T}z}\\ = & 2^{\Upsilon }\cdot {\left|h\right|}^{2}{\left(-1\right)}^{B_{1}+{\left(B{|}_{\Upsilon +1}^{m}\right)}^{T}z}, \end{array} \end{array}$$

where $B_{1}=e_{1}^{T}B$ and $z\in F_{2}^{m-\Upsilon }$. To sum up, $F_{A}\left({\tilde{\Xi }}_{\chi ,z}^{\left(1\right)}\right)$ takes the value of $2^{\Upsilon }\cdot {\left|h\right|}^{2}\cdot {\left(-1\right)}^{B_{1}+{\left(B{|}_{\Upsilon +1}^{m}\right)}^{T}z}$ if and only if ${\tilde{\Xi }}_{\chi ,z}^{\left(1\right)}=\tilde{I_{\chi }}z+{\tilde{p}}_{\chi ,1}$.

Next, we calculate the distribution of $Z({\tilde{\Xi }}_{\chi ,z}^{\left(1\right)})$: we expand $Z({\tilde{\Xi }}_{\chi ,z}^{\left(1\right)})$ into two parts:

(23) $$\begin{array}{c} \begin{array}{cc} Z_{1}\left({\tilde{\Xi }}_{\chi ,z}^{\left(1\right)}\right) & =n_{1}^{H}E\left(I_{\chi }e_{1},\tilde{I_{\chi }}z+{\tilde{p}}_{\chi ,1}\right)n_{1}\\ & =i^{{\left(I_{\chi }e_{1}\right)}^{T}\cdot \sum_{z\in F_{2}^{m-r}}\left(\tilde{I_{\chi }}z+I_{\chi }{\hat{P}}_{1}\right)}\sum_{w\in F_{2}^{m}}\bar{n_{1}\left(w+I_{\chi }e_{1}\right)}\cdot {\left(-1\right)}^{w^{T}\cdot \sum_{z\in F_{2}^{m-r}}\left(\tilde{I_{\chi }}z+I_{\chi }{\hat{P}}_{1}\right)}\cdot n\left(w\right). \end{array} \end{array}$$

Therefore, the variance of $Z_{1}\left({\tilde{\Xi }}_{\chi ,z}^{\left(1\right)}\right)$ is $2^{m}\cdot N_{0}^{2}$. Following the same rule, $Z_{2}$ turns into

(24) $$\begin{array}{c} \begin{array}{cc} Z_{2}\left({\tilde{\Xi }}_{\chi ,z}^{\left(1\right)}\right)= & h\cdot \left[C_{\hat{P},B,I_{\chi }}^{H}E\left(I_{\chi }e_{1},\tilde{I_{\chi }}z+{\tilde{p}}_{\chi ,1}\right)n+n^{H}E\left(I_{\chi }e_{1},\tilde{I_{\chi }}z+{\tilde{p}}_{\chi ,1}\right)C_{\hat{P},B,I_{\chi }}\right]\\ = & h\cdot i^{{\left(I_{\chi }e_{1}\right)}^{T}\cdot \left(\tilde{I_{\chi }}z+I_{\chi }{\hat{P}}_{1}\right)}\sum_{w\in F_{2}^{m}}\bar{C_{\hat{P},B,I_{\chi }}\left(w+I_{\chi }e_{1}\right)}\cdot {\left(-1\right)}^{w^{T}\cdot \left(\tilde{I_{\chi }}z+I_{\chi }{\hat{P}}_{1}\right)}\cdot n\left(w\right)\\ + & h\cdot i^{{\left(I_{\chi }e_{1}\right)}^{T}\cdot \left(\tilde{I_{\chi }}z+I_{\chi }{\hat{P}}_{1}\right)}\sum_{w\in F_{2}^{m}}\bar{n\left(w+I_{\chi }e_{1}\right)}\cdot {\left(-1\right)}^{w^{T}\cdot \left(\tilde{I_{\chi }}z+I_{\chi }{\hat{P}}_{1}\right)}\cdot C_{\hat{P},B,I_{\chi }}\left(w\right). \end{array} \end{array}$$

According to (24), the mean of variable $Z_{2}\left({\tilde{\Xi }}_{\chi ,z}^{\left(1\right)}\right)$ is 0 and the variance is $2\cdot 2^{m}{\left|h\right|}^{2}N_{0}$. We denote the notation $\sigma_{F,1}^{2}=2^{m-1}\left({2|h|}^{2}N_{0}+N_{0}^{2}\right)$. Since the distribution of $Z({\bar{\bar{\Xi }}}_{\chi ,z}^{\left(1\right)})$ is the same with $Z({\tilde{\Xi }}_{\chi ,z}^{\left(1\right)})$, we have $Z\left({\tilde{\Xi }}_{\chi ,z}^{\left(1\right)}\right)∼\mathrm{CN}\left(0,2\sigma_{F,1}^{2}\right)$, $Z\left({\bar{\bar{\Xi }}}_{\chi ,z}^{\left(1\right)}\right)∼\mathrm{CN}\left(0,2\sigma_{F,1}^{2}\right)$ and $F_{A}\left({\tilde{\Xi }}_{\chi ,z}^{\left(1\right)}\right)+Z\left({\tilde{\Xi }}_{\chi ,z}^{\left(1\right)}\right)∼\mathrm{CN}(2^{\Upsilon }\cdot {\left|h\right|}^{2}\cdot {\left(-1\right)}^{B_{1}+{\left(B{|}_{\Upsilon +1}^{m}\right)}^{T}z},2\sigma_{F,1}^{2})$.

Based on above distribution, the first term of the last equation in (19) can be approximated as:

(25) $$\begin{array}{c} \begin{array}{cc} \; & \mathrm{Pr}\left({\left|h\right|}^{2}C_{\hat{P},B,I_{\chi }}^{H}E\left(I_{\chi }e_{1},{\tilde{\Xi }}_{\chi ,z}^{\left(1\right)}\right)C_{\hat{P},B,I_{\chi }}+Z\left({\tilde{\Xi }}_{\chi ,z}^{\left(1\right)}\right)>\left|Z({\bar{\bar{\Xi }}}_{\chi ,z}^{\left(1\right)})\right|\right)\\ = & 1-\mathrm{Pr}\left(Z_{1}\left({\bar{\bar{\Xi }}}_{\chi ,z}^{\left(1\right)}\right)+Z_{2}\left({\bar{\bar{\Xi }}}_{\chi ,z}^{\left(1\right)}\right)-Z\left({\tilde{\Xi }}_{\chi ,z}^{\left(1\right)}\right)\geq {\left|h\right|}^{2}C_{\hat{P},B,I_{\chi }}^{H}E\left(I_{\chi }e_{1},{\tilde{\Xi }}_{\chi ,z}^{\left(1\right)}\right)C_{\hat{P},B,I_{\chi }}\right)\cdot \mathrm{Pr}\left(Z_{1}\left({\bar{\bar{\Xi }}}_{\chi ,z}^{\left(1\right)}\right)+Z_{2}\left({\bar{\bar{\Xi }}}_{\chi ,z}^{\left(1\right)}\right)>0\right)\\ & -\mathrm{Pr}\left(-Z_{1}\left({\bar{\bar{\Xi }}}_{\chi ,z}^{\left(1\right)}\right)-Z_{2}\left({\bar{\bar{\Xi }}}_{\chi ,z}^{\left(1\right)}\right)-Z\left({\tilde{\Xi }}_{\chi ,z}^{\left(1\right)}\right)\geq {\left|h\right|}^{2}C_{\hat{P},B,I_{\chi }}^{H}E\left(I_{\chi }e_{1},{\tilde{\Xi }}_{\chi ,z}^{\left(1\right)}\right)C_{\hat{P},B,I_{\chi }}\right)\cdot \mathrm{Pr}\left(Z_{1}\left({\bar{\bar{\Xi }}}_{\chi ,z}^{\left(1\right)}\right)+Z_{2}\left({\bar{\bar{\Xi }}}_{\chi ,z}^{\left(1\right)}\right)<0\right)\\ = & 1-2\mathrm{Pr}\left(Z_{1}\left({\bar{\bar{\Xi }}}_{\chi ,z}^{\left(1\right)}\right)+Z_{2}\left({\bar{\bar{\Xi }}}_{\chi ,z}^{\left(1\right)}\right)-Z\left({\tilde{\Xi }}_{\chi ,z}^{\left(1\right)}\right)\geq {\left|h\right|}^{2}C_{\hat{P},B,I_{\chi }}^{H}E\left(I_{\chi }e_{1},{\tilde{\Xi }}_{\chi ,z}^{\left(1\right)}\right)C_{\hat{P},B,I_{\chi }}\right)\cdot \mathrm{Pr}\left(Z_{1}\left({\bar{\bar{\Xi }}}_{\chi ,z}^{\left(1\right)}\right)+Z_{2}\left({\bar{\bar{\Xi }}}_{\chi ,z}^{\left(1\right)}\right)>0\right)\\ \overset{\left(d\right)}{\geq } & 1-Q\left(\frac{2^{\Upsilon }{\left|h\right|}^{2}\cdot {\left(-1\right)}^{B_{1}+{\left(B{|}_{\Upsilon +1}^{m}\right)}^{T}z}}{2\sigma_{F,1}}\right), \end{array} \end{array}$$

where (d) depends on $Z_{1}\left({\bar{\bar{\Xi }}}_{\chi ,z}^{\left(1\right)}\right)+Z_{2}\left({\bar{\bar{\Xi }}}_{\chi ,z}^{\left(1\right)}\right)-Z\left({\tilde{\Xi }}_{\chi ,z}^{\left(1\right)}\right)∼N\left(0,4\sigma_{F,1}^{2}\right)$. We then compute the latter term of (19) as

(26) $$\begin{array}{c} \begin{array}{cc} \; & \mathrm{Pr}\left({\left|h\right|}^{2}C_{\hat{P},B,I_{\chi }}^{H}E\left(I_{\chi }e_{1},{\tilde{\Xi }}_{\chi ,z}^{\left(1\right)}\right)C_{\hat{P},B,I_{\chi }}+Z\left({\tilde{\Xi }}_{\chi ,z}^{\left(1\right)}\right)>0\right)\\ = & \mathrm{Pr}\left(Z\left({\tilde{\Xi }}_{\chi ,z}^{\left(1\right)}\right)>-{\left|h\right|}^{2}C_{\hat{P},B,I_{\chi }}^{H}E\left(I_{\chi }e_{1},{\tilde{\Xi }}_{\chi ,z}^{\left(1\right)}\right)C_{\hat{P},B,I_{\chi }}\right)\\ = & \mathrm{Pr}\left(Z\left({\tilde{\Xi }}_{\chi ,z}^{\left(1\right)}\right)>-2^{\Upsilon }{\left|h\right|}^{2}\cdot {\left(-1\right)}^{B_{1}+{\left(B{|}_{\Upsilon +1}^{m}\right)}^{T}z}\right)\\ = & Q\left(-\frac{2^{\Upsilon }{\left|h\right|}^{2}\cdot {\left(-1\right)}^{B_{1}+{\left(B{|}_{\Upsilon +1}^{m}\right)}^{T}z}}{\sqrt{2}\sigma_{F,1}}\right). \end{array} \end{array}$$

By substituting (25) and (26) into (18), we finally get

(27) $$\begin{array}{c} \begin{array}{cc} \; & \prod_{{\left[{\bar{\bar{p}}}_{\chi ,1}^{\left(j\right)}\right]}_{j=1}^{2^{\Upsilon -1}}}\mathrm{Pr}\left(\left|F\left(\tilde{I_{\chi }}z+{\tilde{p}}_{\chi ,1}\right)\right|>\left|F\left(\tilde{I_{\chi }}z+{\bar{\bar{p}}}_{\chi ,1}^{\left(j\right)}\right)\right|\right)\\ \geq & {\left[Q\left(-\frac{2^{\Upsilon }{\left|h\right|}^{2}\cdot {\left(-1\right)}^{B_{1}+{\left(B{|}_{\Upsilon +1}^{m}\right)}^{T}z}}{\sqrt{2}\sigma_{F,1}}\right)\cdot Q\left(-\frac{2^{\Upsilon }{\left|h\right|}^{2}\cdot {\left(-1\right)}^{B_{1}+{\left(B{|}_{\Upsilon +1}^{m}\right)}^{T}z}}{2\sigma_{F,1}}\right)\right]}^{2^{\Upsilon }-1}. \end{array} \end{array}$$

Equation (27) is consistent with (13) in Lemma 1, so Lemma 1 holds for r=1.

(2) When 2≤r≤Υ: Algorithm 1’s input signal becomes

(28) $$\begin{array}{c} \begin{array}{cc} y_{r} & =\lambda_{PRM,\varpi }^{\left(r\right)}\cdot y_{r-1}\\ & =C_{\hat{P},B,I_{\chi }}+\prod_{i=1}^{r-1}\lambda_{PRM,\varpi }^{\left(i\right)}\cdot n\\ & =C_{\hat{P},B,I_{\chi }}+\frac{1}{2^{i-1}}\prod_{i=1}^{r-1}\left(I_{N}+\varpi_{i}\cdot E\left[I_{\chi }e_{i},I_{\chi }{\hat{P}}_{i}\right]\right)\cdot n. \end{array} \end{array}$$

By substituting (28) into (16), we have $\sigma_{F,r}^{2}=2^{m-1}\left[{2|h|}^{2}\frac{N_{0}}{2^{r-1}}+{\left(\frac{N_{0}}{2^{r-1}}\right)}^{2}\right]$. Thus, $Z\left({\tilde{\Xi }}_{\chi ,z}^{\left(r\right)}\right)∼\mathrm{CN}\left(0,2\sigma_{F,r}^{2}\right)$, and $Z\left({\bar{\bar{\Xi }}}_{\chi ,z}^{\left(r\right)}\right)∼\mathrm{CN}\left(0,2\sigma_{F,r}^{2}\right)$. Furthermore, we get $F_{A}\left({\tilde{\Xi }}_{\chi ,z}^{\left(r\right)}\right)+Z\left({\tilde{\Xi }}_{\chi ,z}^{\left(r\right)}\right)∼\mathrm{CN}(2^{\Upsilon }\cdot {\left|h\right|}^{2}{\left(-1\right)}^{B_{r}+{\left(B{|}_{\Upsilon +1}^{m}\right)}^{T}z},2\sigma_{F,r}^{2})$. The derivation process is similar to r=1, and we omit it here. The result is consistent with (13) and (14) in Lemma 1.

The PRM sequence is reconstructed successfully if the estimations for all layers are correct. Therefore, Algorithm 1 has a successful detection probability as follows:

(29) $$\overset{ˇ}{P}\left(\Upsilon ,N_{0}|h\right)=\prod_{r=1}^{\Upsilon }\mathrm{Pr}\left(P^{\left(r\right)}|P^{\left(1,\cdots ,r-1\right)},h\right).$$

Taking the channel coefficient *h* into account, we get the following equation

(30) $$P_{succ}=\int_{h}f_{\mathrm{CN}}\left(h;0,1\right)\cdot \overset{ˇ}{P}\left(\Upsilon ,N_{0}|h\right)dh,$$

Thus, the proof of Theorem 1 is complete. □

**Figure 2:**
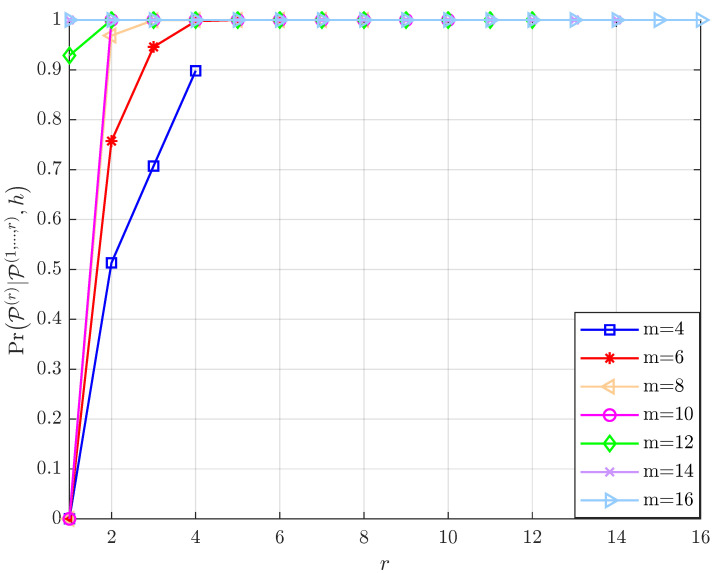
Given *m*, $N_{0}=10$ and Υ, the variation of $\mathrm{Pr}\left(P^{\left(r\right)}|P^{\left(1,\cdots ,r-1\right)},h\right)$ with r.

**Figure 3:**
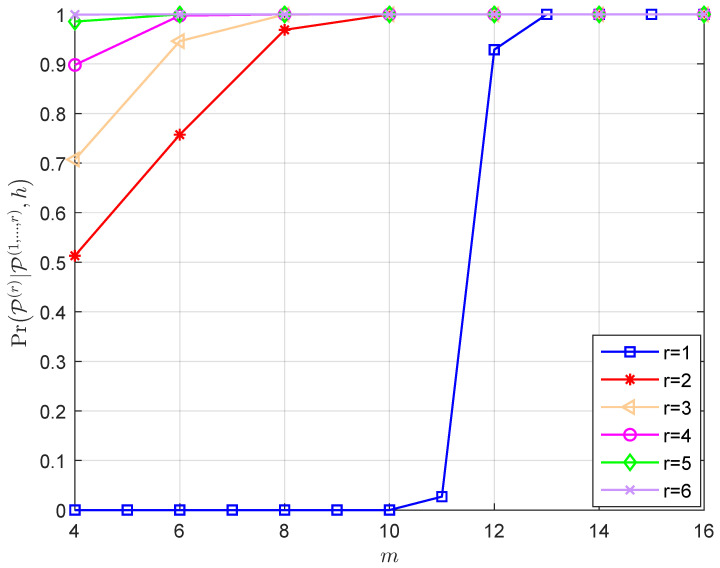
Given r, $N_{0}=10$ and Υ, the variation of $\mathrm{Pr}\left(P^{\left(r\right)}|P^{\left(1,\cdots ,r-1\right)},h\right)$ with *m*.

**Figure 4:**
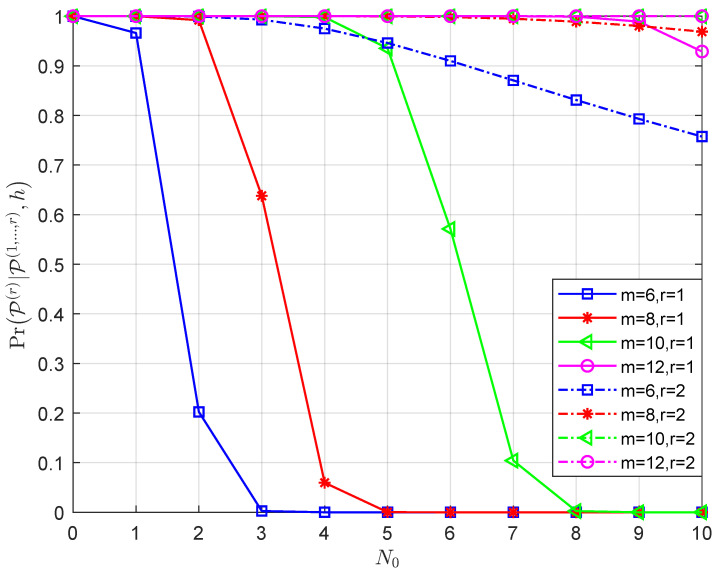
Given r, *m* and Υ, the variation of $\mathrm{Pr}\left(P^{\left(r\right)}|P^{\left(1,\cdots ,r-1\right)},h\right)$ with $N_{0}$.

**Figure 5:**
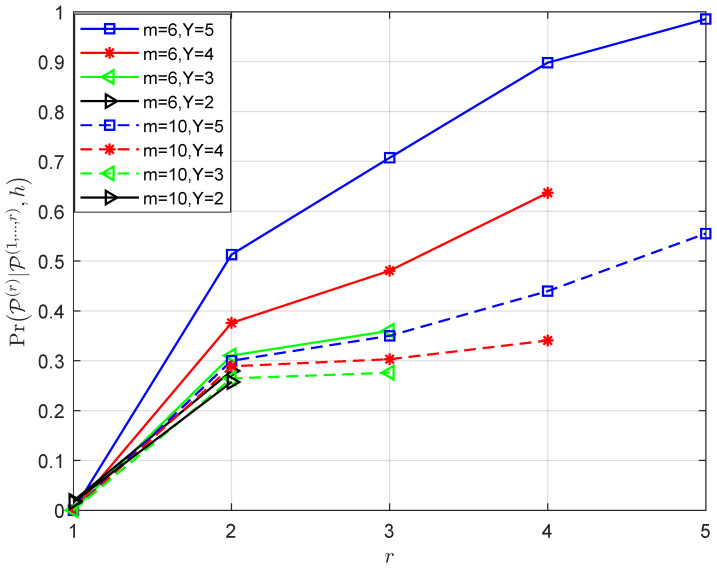
Given $N_{0}$, *m* and Υ, the variation of $\mathrm{Pr}\left(P^{\left(r\right)}|P^{\left(1,\cdots ,r-1\right)},h\right)$ with r.

The factors that affect the efficiency of the layer-by-layer PRM projection algorithm are discussed below. According to Lemma 1, the conditional probability $\mathrm{Pr}\left(P^{\left(r\right)}|P^{\left(1,\cdots ,r-1\right)},h\right)$ of the vector ${\hat{P}}_{r}$ is related to three factors, namely Υ, r, *m* and $N_{0}$. Since it is challenging to mathematically express the relationship between them, we fix two elements and track the changes in the conditional probability as a result of changing the third factor. In accordance with Figure 2, Figure 3, Figure 4 and Figure 5, the following results are obtained:

- Assume that the PRM code is of full rank with Υ=m, when *m* and $N_{0}$ are given, as r increases from 1 to *m*, the value of $\mathrm{Pr}\left(P^{\left(r\right)}|P^{\left(1,\cdots ,r-1\right)},h\right)$ increases (Figure 2). The results illustrate the properties of the projection algorithm: if the first few layers are error-free, then the remaining layers are highly likely to be recovered correctly. Additionally, a further conclusion can be drawn by observing the above proof that $n_{r}∼\mathrm{CN}\left(0,\frac{N_{0}}{2^{r-1}}\right)$ indicates that the noise power in each layer decreases exponentially as r increases, leading to the successful recovery probability of the vector increasing layer by layer. Overall, the recovery reliability of the first few layers of the layer-by-layer PRM projection algorithm is crucial to its performance. This motivates us to increase the number of candidate paths in the first few layers to obtain better detection.
- As shown in Figure 3, when r, Υ and $N_{0}$ are given, $\mathrm{Pr}\left(P^{\left(r\right)}|P^{\left(1,\cdots ,r-1\right)},h\right)$ increases as *m* increases, which shows that we can improve the performance of the PRM sequence detection algorithm by increasing the length of the PRM sequence.
- Figure 4 details the variation of $\mathrm{Pr}\left(P^{\left(r\right)}|P^{\left(1,\cdots ,r-1\right)},h\right)$ with $N_{0}$ for fixed r, *m* and Υ. It is clear that as $N_{0}$ increases, the signal-to-noise ratio (SNR) decreases, resulting in $\mathrm{Pr}\left(P^{\left(r\right)}|P^{\left(1,\cdots ,r-1\right)},h\right)$ decreasing consequently.
- Figure 5 gives the variation of $\mathrm{Pr}\left(P^{\left(r\right)}|P^{\left(1,\cdots ,r-1\right)},h\right)$ with r for fixed $N_{0}$, *m* and Υ: the larger Υ indicates the better performance under the same *m*, and this result is consistent with the fact that a greater signal power will result in a greater signal-to-noise ratio. Furthermore, the larger the *m*, the worse the performance under the same layer r, indicating that the closer Υ to *m*, the better the performance will be.

### 4.2. Successful Recovery Probability of the Iterative List PRM Projection Algorithm

In this section, Lemma 2 and Theorem 2 analyze the performance of the iterative list PRM projection algorithm in a multi-user scenario.

**Lemma 2.**  *Suppose that the input signal of Algorithm 2 contains a linear combination of all $K_{a}$ active users’ sequences. We let these sequences be detected in the order of $\left(\rho_{1},\rho_{2},\cdots ,\rho_{K_{a}}\right)$. We denote the event “the $\rho_{k}$-th recovered sequence has been successfully detected” as $B_{\rho_{k}}$ and the event “the sequences with order $\left(\rho_{1},\cdots ,\rho_{k-1}\right)$ have been successfully detected and have been subtracted from the received signal” as $B_{\rho_{1},\cdots ,\rho_{k-1}}$. Thus, given the channel parameters $\underline{h}=\left[h_{1},\cdots ,h_{K_{a}}\right]$ and the event $B_{\rho_{1},\cdots ,\rho_{k-1}}$, the chance of the event $B_{\rho_{1},\cdots ,\rho_{k-1}}$ occurs in the first iteration of Algorithm 2 can be approximated as*

(31) $$\begin{array}{c} \begin{array}{cc} \; & \mathrm{Pr}\left(B_{\rho_{k}}|B_{\rho_{1},\cdots ,\rho_{k-1}},h\right)=\mathrm{Pr}\left(P_{\rho_{k}}^{\left(1\right)}|\underline{h}\right)\cdot \overset{ˇ}{P}\left(\Upsilon_{\rho_{k}}-1,N_{0}^{\left(\Upsilon_{\rho_{k}}-1\right)}|\underline{h}\right). \end{array} \end{array}$$

*In (31), $\mathrm{Pr}\left(P_{\rho_{k}}^{\left(1\right)}|\underline{h}\right)$ refers to the probability of ${\hat{P}}_{\rho_{k}}e_{1}$ being successfully recovered under the condition $\underline{h}$, which is expressed as*

(32) $$\begin{array}{c} \begin{array}{cc} \; & \mathrm{Pr}\left(P_{\rho_{k}}^{\left(1\right)}|\underline{h}\right)\\ = & \prod_{j=1}^{2^{\Upsilon_{\rho_{k}}}-1}Q\left(-\frac{2^{\Upsilon_{\rho_{k}}}{\left|h_{\rho_{k}}\right|}^{2}+\sum_{\rho_{k+1}\leq i\leq \rho_{K_{a}}}\left(n_{\rho_{k},i}\cdot 2^{\Upsilon_{\rho_{i}}}|h_{i}{|}^{2}\cdot -n_{i,j}\cdot 2^{\Upsilon_{\rho_{i}}}{\left|h_{i}\right|}^{2}\right)}{\sqrt{2}\sigma_{\Lambda ,1}}\right)\\ & \;\cdot Q{\left(-\frac{2^{\Upsilon_{\rho_{k}}}{\left|h_{\rho_{k}}\right|}^{2}}{\sigma_{\Lambda ,1}}\right)}^{2^{\Upsilon_{\rho_{k}}}-1}, \end{array} \end{array}$$

*where the notation $n_{\rho_{k},i}$ indicates the number of intersections of positions corresponding to non-zero F(·) for the i-th detected sequence and $\rho_{k}$-th detected sequence. The notation $n_{i,j}$ represents the number of intersections of positions corresponding to non-zero F(·) for the i-th detected sequence and the j-th coset of the set related to non-zero F(·) for the first layer recovery of the $\rho_{k}$-th detected sequence. Additionally, the variance in (32) is*

(33) $$\sigma_{\Lambda ,1}^{2}=\sum_{\rho =\rho_{k}}^{\rho_{K_{a}}}\left[\sum_{\rho^{\prime }=\rho_{k}and\rho^{\prime }\neq \rho }^{\rho_{K_{a}}}{\left|h_{\rho }^{H}\cdot h_{\rho^{\prime }}\right|}^{2}\cdot \sigma_{\mu ,1}^{2}+2^{m-1}\cdot \left(\sum_{\rho =\rho_{k}}^{\rho_{K_{a}}}2{\left|h_{\rho }\right|}^{2}N_{0}+N_{0}^{2}\right)\right],$$

*in which μ is the inner product created by two PRM sequences of length-$2^{m}$, and $\sigma_{\mu ,1}^{2}$ denotes the variance of the inner product. The second component $\overset{ˇ}{P}\left(\Upsilon_{\rho_{1}}-1,N_{0}^{\left(\Upsilon_{\rho_{k}}-1\right)}|h\right)$ in Equation (31) represents the matrix ${\hat{P}}^{\Upsilon_{\rho_{k}}-1}$’s likelihood of a successful recovery given h, where ${\hat{P}}^{\Upsilon_{\rho_{k}}-1}$ denotes the submatrix of the symmetric matrix ${\hat{P}}_{\rho_{k}}$ with rank $\left(\Upsilon_{\rho_{k}}-1\right)$ for order $\rho_{k}$. Additionally, the notation $N_{0}^{\left(\Upsilon_{\rho_{k}}-1\right)}=\frac{1}{2}(\sum_{\rho =\rho_{k+1}}^{\rho_{K_{a}}}|h_{\rho }{|}^{2}+N_{0})$.*

**Theorem 2.**  *Suppose that the input signal of Algorithm 2 contains a linear combination of all $K_{a}$ active users’ sequences. We denote each sequence by the specific notation k ($1\leq k\leq K_{a}$) and let these sequences be detected in the order of $\left(\rho_{1},\rho_{2},\cdots ,\rho_{K_{a}}\right)$. Then, given the channel parameter $\underline{h}$, the probability that Algorithm 2 can successfully detect the sequence ρ$\left(1\leq \rho \leq K_{a}\right)$ in the first iteration is*

(34) $$\begin{array}{c} \begin{array}{cc} \; & {\overset{ˇ}{\overset{ˇ}{P}}}_{\rho }\left(K_{a},m,N_{0}|\underline{h}\right)\\ = & \mathrm{Pr}\left(B_{\rho }|\underline{h}\right)+\sum_{k=2}^{K_{a}}\left\{\sum_{\left(\rho_{1},\cdots ,\rho_{k-1}\right)\in N_{k}}\left[\left(\prod_{k^{\prime }=1}^{k-1}\mathrm{Pr}\left(B_{\rho_{k^{\prime }}}|B_{\rho_{1},\cdots ,\rho_{k^{\prime }-1}},\underline{h}\right)\right)\cdot \mathrm{Pr}\left(B_{\rho }|B_{\rho_{1},\cdots ,\rho_{k-1}},\underline{h}\right)\right]\right\}, \end{array} \end{array}$$

*where the set $N_{k}$ contains all permutations of (k−1) indexes of order from the set $K_{a}\setminus \rho$. Furthermore, by taking the channel parameters into consideration, the average probability of successful detection for the first iteration of the iterative PRM sequence can be approximated as*

(35) $$\begin{array}{c} \begin{array}{c} {\overset{ˇ}{\overset{ˇ}{P}}}_{\mathrm{ave}}\left(K_{a},m,N_{0}|\underline{h}\right)=\frac{1}{K_{a}}\int_{\underline{h}}\left\{\prod_{\rho =1}^{K_{a}}f_{\mathrm{CN}}\left(h_{\rho };0,1\right)\cdot \sum_{\rho =1}^{K_{a}}{\overset{ˇ}{\overset{ˇ}{P}}}_{\rho }\left(K_{a},m,N_{0}|\underline{h}\right)\right\}. \end{array} \end{array}$$

**Proof.**  For convenience, two users are assumed to be active in the system. Since users are not provided with IDs in the URA scenario, we can deduce the theoretical results of successful detection probabilities with the help of the detection order between sequences; we denote two sequences as *a* and *b*, respectively, and the detection order between them is represented by $\rho_{a}$ and $\rho_{b}$. At this point, the input signal of the algorithm is rewritten as a linear superposition of the two users of the form: $y=h_{a}C_{{\hat{P}}_{a},B_{a},I_{\chi ,a}}+h_{b}C_{{\hat{P}}_{b},B_{b},I_{\chi ,b}}+n.$ The rank of PRM codes $C_{{\hat{P}}_{a},B_{a},I_{\chi ,a}}$ and $C_{{\hat{P}}_{b},B_{b},I_{\chi ,b}}$ are noted as $\Upsilon_{a}$ and $\Upsilon_{b}$, respectively. According to the expansion of (16), the $F_{M}$ can be expanded regarding the r-layer as

(36) $$\begin{array}{c} \begin{array}{cc} F_{M}\left(f_{r}\right)= & {\left(h_{a}C_{{\hat{P}}_{a},B_{a},I_{\chi ,a}}+h_{b}C_{{\hat{P}}_{b},B_{b},I_{\chi ,b}}+n\right)}^{H}\cdot E\left(I_{\chi }e_{r},f_{r}\right)\cdot \left(h_{a}C_{{\hat{P}}_{a},B_{a},I_{\chi ,a}}+h_{b}C_{{\hat{P}}_{b},B_{b},I_{\chi ,b}}+n\right)\\ = & \underset{F_{a}\left(f_{r}\right)}{\underset{︸}{|h_{a}{|}^{2}C_{{\hat{P}}_{a},B_{a},I_{\chi ,a}}^{H}E\left(I_{\chi }e_{r},f_{r}\right)C_{{\hat{P}}_{a},B_{a},I_{\chi ,a}}}}+\underset{F_{b}\left(f_{r}\right)}{\underset{︸}{|h_{b}{|}^{2}C_{{\hat{P}}_{b},B_{b},I_{\chi ,b}}^{H}E\left(I_{\chi }e_{r},f_{r}\right)C_{{\hat{P}}_{b},B_{b},I_{\chi ,b}}}}\\ \; & +\underset{Z_{ab,1}\left(f_{r}\right)}{\underset{︸}{h_{a}^{H}\cdot h_{b}C_{{\hat{P}}_{a},B_{a},I_{\chi ,a}}^{H}E\left(I_{\chi }e_{r},f_{r}\right)C_{{\hat{P}}_{b},B_{b},I_{\chi ,b}}}}+\underset{Z_{ab,2}\left(f_{r}\right)}{\underset{︸}{h_{a}\cdot h_{b}^{H}C_{{\hat{P}}_{b},B_{b},I_{\chi ,b}}^{H}E\left(I_{\chi }e_{r},f_{r}\right)C_{{\hat{P}}_{a},B_{a},I_{\chi ,a}}}}\\ \; & +\underset{X_{a,1}\left(f_{r}\right)}{\underset{︸}{h_{a}\left[C_{{\hat{P}}_{a},B_{a},I_{\chi ,a}}^{H}E\left(I_{\chi }e_{r},f_{r}\right)n+n^{H}E\left(I_{\chi }e_{r},f_{r}\right)C_{{\hat{P}}_{a},B_{a},I_{\chi ,a}}\right]}}\\ \; & +\underset{X_{a,2}\left(f_{r}\right)}{\underset{︸}{h_{b}\left[C_{{\hat{P}}_{b},B_{b},I_{\chi ,b}}^{H}E\left(I_{\chi }e_{r},f_{r}\right)n+n^{H}E\left(I_{\chi }e_{r},f_{r}\right)C_{{\hat{P}}_{b},B_{b},I_{\chi ,b}}\right]}}+\underset{N(f_{r})}{\underset{︸}{n^{H}E\left(I_{\chi }e_{r},f_{r}\right)n}}. \end{array} \end{array}$$

For ease of use, we make $\left(Z_{ab,1}+Z_{ab,2}+X_{a,1}+X_{a,2}+N\right)\left(f_{r}\right)=\Lambda \left(f_{r}\right)$.

We first assess the performance of the first-layer (r=1) detection for the $\rho_{a}$-th sequence: suppose in the first iteration of Algorithm 2, active users are detected successively according to the order $\left(\rho_{a},\rho_{b}\right)$. In the following, we write the probability of the event $P_{\rho_{a}}^{\left(1\right)}$ as form

(37) $$\begin{array}{c} \begin{array}{cc} \mathrm{Pr}\left(P_{\rho_{a}}^{\left(1\right)}|h\right) & =\mathrm{Pr}\left(\sum_{z\in F_{2}^{m-\Upsilon_{a}}}\left|F\left({\tilde{I}}_{\chi ,a}z+{\tilde{p}}_{\chi_{a},1}\right)\right|>\max\sum_{z\in F_{2}^{m-\Upsilon_{a}}}\left|F\left({\tilde{I}}_{\chi ,a}z+{\bar{\bar{p}}}_{\chi_{a},1}\right)\right|\right)\\ \; & =\prod_{{\left[{\bar{\bar{p}}}_{\chi_{a},1}^{\left(j\right)}\right]}_{j}^{2^{\Upsilon_{a}}-1}}\mathrm{Pr}\left(\sum_{z\in F_{2}^{m-\Upsilon_{a}}}\left|F\left({\tilde{I}}_{\chi ,a}z+{\tilde{p}}_{\chi_{a},1}\right)\right|>\sum_{z\in F_{2}^{m-\Upsilon_{a}}}\left|F\left({\tilde{I}}_{\chi ,a}z+{\bar{\bar{p}}}_{\chi_{a},1}^{\left(j\right)}\right)\right|\right). \end{array} \end{array}$$

We omit the subscript M and record it as F(·) in this section. As seen in (37), the present situation is more complicated than the single-user scenario. One of the problems is that the $2^{m-\Upsilon_{a}}$ jointly summed events are no longer independent and no longer obey the same distribution. In addition, for the property of the PRM sequence, the vector $\left({\tilde{I}}_{\chi ,a}z+{\tilde{p}}_{\chi_{a},1}\right)$ in (37) is specific to the $\rho_{a}$-th detected sequences, and the $\rho_{b}$-th sequence utilizes a PRM whose rank $\Upsilon_{b}$ is not necessarily equivalent to $\Upsilon_{a}$; therefore, the analysis requires a detailed consideration of the interference of the other user. For simplicity, we first approximate a single term in (37) as follows:

(38) $$\begin{array}{c} \begin{array}{cc} \; & \mathrm{Pr}\left(\sum_{z\in F_{2}^{m-\Upsilon_{a}}}\left(\left|\left(F_{a}+\Lambda \right)\left({\tilde{\Xi }}_{\chi_{a},z}^{\left(1\right)}\right)\right|+\left|F_{b}\left({\tilde{\Xi }}_{\chi_{a},z}^{\left(1\right)}\right)\right|\right)\geq \sum_{z\in F_{2}^{m-\Upsilon_{a}}}\left(\left|F_{b}\left({\bar{\bar{\Xi }}}_{\chi_{a},z}^{\left(j,1\right)}\right)\right|+\left|\Lambda ({\bar{\bar{\Xi }}}_{\chi_{a},z}^{\left(j,1\right)})\right|\right)\right)\\ \overset{\left(e\right)}{=} & \mathrm{Pr}\left(\left|\left(F_{a}+\Lambda \right)\left({\tilde{\Xi }}_{\chi_{a},z}^{\left(1\right)}\right)\right|+\sum_{z\in F_{2}^{m-\Upsilon_{a}}}\left|F_{b}\left({\tilde{\Xi }}_{\chi_{a},z}^{\left(1\right)}\right)\right|\geq \left|\Lambda ({\bar{\bar{\Xi }}}_{\chi_{a},z}^{\left(j,1\right)})\right|+\sum_{z\in F_{2}^{m-\Upsilon_{a}}}\left|F_{b}\left({\bar{\bar{\Xi }}}_{\chi_{a},z}^{\left(j,1\right)}\right)\right|\right)\\ \overset{\left(f\right)}{\geq } & \mathrm{Pr}\left(\left(F_{a}+\Lambda \right)\left({\tilde{\Xi }}_{\chi_{a},z}^{\left(1\right)}\right)+\sum_{z\in F_{2}^{m-\Upsilon_{a}}}\left|F_{b}\left({\tilde{\Xi }}_{\chi_{a},z}^{\left(1\right)}\right)\right|\geq \left|\Lambda ({\bar{\bar{\Xi }}}_{\chi_{a},z}^{\left(j,1\right)})\right|+\sum_{z\in F_{2}^{m-\Upsilon_{a}}}\left|F_{b}\left({\bar{\bar{\Xi }}}_{\chi_{a},z}^{\left(j,1\right)}\right)\right|\right)\cdot \mathrm{Pr}\left(\left(F_{a}+\Lambda \right)\left({\tilde{\Xi }}_{\chi_{a},z}^{\left(1\right)}\right)>0\right), \end{array} \end{array}$$

where ${\tilde{\Xi }}_{\chi_{a},z}^{\left(1\right)}={\tilde{I}}_{\chi ,a}z+{\tilde{p}}_{\chi_{a},1}$, ${\bar{\bar{\Xi }}}_{\chi_{a},z}^{\left(j,1\right)}={\tilde{I}}_{\chi ,a}z+{\bar{\bar{p}}}_{\chi_{a},1}^{\left(j\right)}$ (different from (19), the superscript “*j*” can not be removed from ${\bar{\bar{\Xi }}}_{\chi_{a},z}^{\left(j,1\right)}$ since the events in joint multiply from (37) are not independent anymore) and ${\bar{\bar{\Xi }}}_{\chi_{a},z}^{\left(j,1\right)}$ is the *j*-th coset of the set ${\tilde{\Xi }}_{\chi_{a},z}^{\left(1\right)}$ ($1\leq j\leq 2^{\Upsilon_{a}}-1$). It is known that $F_{a}\left({\tilde{\Xi }}_{\chi_{a},z}^{\left(1\right)}\right)$ take non-zero values for the set ${\tilde{\Xi }}_{\chi_{a},z}^{\left(1\right)}$. Additionally, the equal sign (e) in (36) retains one of the terms on both sides owing to the same distribution. The inequality sign (f) follows the formula (18). In the following, we will calculate each of the two probabilities of the result of (38).

We first solve the probability of the latter term. Since we know that $F_{a}\left({\tilde{\Xi }}_{\chi_{a},z}^{\left(1\right)}\right)$ takes a fixed value, it is sufficient to obtain the distribution of $\Lambda ({\tilde{\Xi }}_{\chi_{a},z}^{\left(1\right)})$. Thus, the distribution of $Z_{ab,1}\left({\tilde{\Xi }}_{\chi_{a},z}^{\left(1\right)}\right)$ and $Z_{ab,2}\left({\tilde{\Xi }}_{\chi_{a},z}^{\left(1\right)}\right)$ in $\Lambda ({\tilde{\Xi }}_{\chi_{a},z}^{\left(1\right)})$ can be extended as

(39) $$\begin{array}{c} \begin{array}{cc} Z_{ab,1}\left({\tilde{\Xi }}_{\chi_{a},z}^{\left(1\right)}\right)\; & =h_{a}^{H}\cdot h_{b}\cdot C_{{\hat{P}}_{a},B_{a},I_{\chi ,a}}^{H}E\left(I_{\chi_{a}}e_{r},{\tilde{\Xi }}_{\chi_{a},z}^{\left(1\right)}\right)C_{{\hat{P}}_{b},B_{b},I_{\chi ,b}}\\ & =h_{a}^{H}\cdot h_{b}\cdot i^{{\left(I_{\chi }e_{r}\right)}^{T}{\tilde{\Xi }}_{\chi_{a},z}^{\left(1\right)}}\sum_{w\in F_{2}^{m}}\bar{C_{\hat{P_{a}},B_{a},I_{\chi_{a}}}\left(w+I_{\chi_{a}}e_{r}\right)}\cdot {\left(-1\right)}^{w^{T}\cdot {\tilde{\Xi }}_{\chi_{a},z}^{\left(1\right)}}\cdot C_{\hat{P_{b}},B_{b},I_{\chi_{b}}}\left(w\right)\\ & =h_{a}^{H}\cdot h_{b}\cdot i^{{\left(I_{\chi }\cdot e_{r}+2w\right)}^{T}\cdot {\tilde{\Xi }}_{\chi_{a},z}^{\left(1\right)}}\cdot \mu_{\bar{\rho_{a}},\rho_{b}}^{\left(1\right)} \end{array} \end{array}$$

and

(40) $$\begin{array}{c} \begin{array}{cc} Z_{ab,2}\left({\tilde{\Xi }}_{\chi_{a},z}^{\left(1\right)}\right) & =h_{a}\cdot h_{b}^{H}\cdot C_{{\hat{P}}_{b},B_{b},I_{\chi ,b}}^{H}E\left(I_{\chi_{a}}e_{r},{\tilde{\Xi }}_{\chi_{a},z}^{\left(1\right)}\right)C_{{\hat{P}}_{a},B_{a},I_{\chi ,a}}\\ & =h_{a}\cdot h_{b}^{H}\cdot i^{{\left(I_{\chi }e_{r}\right)}^{T}{\tilde{\Xi }}_{\chi_{a},z}^{\left(1\right)}}\sum_{w\in F_{2}^{m}}\bar{C_{\hat{P_{b}},B_{b},I_{\chi_{b}}}\left(w+I_{\chi_{a}}e_{r}\right)}\cdot {\left(-1\right)}^{w^{T}\cdot {\tilde{\Xi }}_{\chi_{a},z}^{\left(1\right)}}\cdot C_{\hat{P_{a}},B_{a},I_{\chi_{a}}}\left(w\right)\\ & =h_{a}\cdot h_{b}^{H}\cdot i^{{\left(I_{\chi }\cdot e_{r}+2w\right)}^{T}\cdot {\tilde{\Xi }}_{\chi_{a},z}^{\left(1\right)}}\cdot \mu_{\rho_{a},\bar{\rho_{b}}}^{\left(1\right)}, \end{array} \end{array}$$

respectively, where $\mu_{\bar{\rho_{a}},\rho_{b}}^{\left(1\right)}$ and $\mu_{\rho_{a},\bar{\rho_{b}}}^{\left(1\right)}$ are the inner product of two sequences. The inner product is related to the pattern form of the PRM. Since the distribution of the occupancy pattern has equal probability, the mean of the inner product is 0, and we denote its variance as $\sigma_{\mu ,1}^{2}$. Thus, we can obtain that $\Lambda ({\tilde{\Xi }}_{\chi_{a},z}^{\left(1\right)})$ obeys the distribution $N(0,\sigma_{\Lambda ,1}^{2})$, where the variance is

$$\sigma_{\Lambda ,1}^{2}=\left(\right|h_{a}^{H}\cdot h_{b}{|}^{2}+|h_{a}\cdot h_{b}^{H}{|}^{2})\cdot \sigma_{\mu ,1}^{2}+2^{m-1}\left[2(|h_{a}{|}^{2}+|h_{b}{|}^{2})N_{0}+N_{0}^{2}\right].$$

In light of this, the latter term of (38) can be computed as

(41) $$\begin{array}{c} \begin{array}{cc} \; & \mathrm{Pr}\left(\left(F_{a}+\Lambda \right)\left({\tilde{\Xi }}_{\chi_{a},z}^{\left(1\right)}\right)>0\right)\\ = & \mathrm{Pr}\left(\Lambda \left({\tilde{\Xi }}_{\chi_{a},z}^{\left(1\right)}\right)>-F_{a}\left({\tilde{\Xi }}_{\chi_{a},z}^{\left(1\right)}\right)\right)\\ = & \mathrm{Pr}\left(\Lambda \left({\tilde{\Xi }}_{\chi_{a},z}^{\left(1\right)}\right)>-2^{\Upsilon_{a}}{\left|h_{a}\right|}^{2}\cdot {\left(-1\right)}^{B_{a,1}+{\left(B_{a}{|}_{\Upsilon +1}^{m}\right)}^{T}z}\right)\\ \overset{\left(g\right)}{=} & Q\left(-\frac{2^{\Upsilon_{a}}{\left|h_{a}\right|}^{2}}{\sigma_{\Lambda ,1}}\right). \end{array} \end{array}$$

In equal sign (g), we set $z=0^{m-\Upsilon }$; this setting is also extended in the following formulas.

We next solve the probability of the first term in (38), we suppose that the set corresponding to one-zero $F_{b}\left(\cdot \right)$ is noted as ${\tilde{\Xi }}_{\chi_{b},z}^{\left(1\right)}$, and we make $|{\tilde{\Xi }}_{\chi_{a},z}^{\left(1\right)}\cap {\tilde{\Xi }}_{\chi_{b},z}^{\left(1\right)}|=n_{ab}$, obviously, $|{\tilde{\Xi }}_{\chi_{b},z}^{\left(1\right)}|=2^{m-\Upsilon_{b}}$. We further denote $|{\bar{\bar{\Xi }}}_{\chi_{a},z}^{\left(j,1\right)}\cap {\tilde{\Xi }}_{\chi_{b},z}^{\left(1\right)}|=n_{b,j}$ and $n_{ab}+\sum_{j=1}^{2^{\Upsilon_{a}}-1}n_{b,j}=2^{m-\Upsilon_{b}}$. On this basis, the first term of the results of (38) yields the following equation:

(42) $$\begin{array}{c} \begin{array}{cc} \; & \mathrm{Pr}\left(\left(F_{a}+\Lambda \right)\left({\tilde{\Xi }}_{\chi_{a},z}^{\left(1\right)}\right)+\sum_{z\in F_{2}^{m-\Upsilon_{a}}}\left|F_{b}\left({\tilde{\Xi }}_{\chi_{a},z}^{\left(1\right)}\right)\right|\geq \left|\Lambda ({\bar{\bar{\Xi }}}_{\chi_{a},z}^{\left(j,1\right)})\right|+\sum_{z\in F_{2}^{m-\Upsilon_{a}}}\left|F_{b}\left({\bar{\bar{\Xi }}}_{\chi_{a},z}^{\left(j,1\right)}\right)\right|\right)\\ = & \mathrm{Pr}\left(\left(F_{a}+\Lambda \right)\left({\tilde{\Xi }}_{\chi_{a},z}^{\left(1\right)}\right)+n_{ab}\cdot 2^{\Upsilon_{b}}|h_{b}{|}^{2}\geq \left|\Lambda ({\bar{\bar{\Xi }}}_{\chi_{a},z}^{\left(j,1\right)})\right|+n_{b,j}\cdot 2^{\Upsilon_{b}}{\left|h_{b}\right|}^{2}2^{\Upsilon_{b}}\right)\\ = & 1-\mathrm{Pr}\left(\Lambda \left({\bar{\bar{\Xi }}}_{\chi_{a},z}^{\left(j,1\right)}\right)-\Lambda \left({\tilde{\Xi }}_{\chi_{a},z}^{\left(1\right)}\right)\geq 2^{\Upsilon_{a}}|h_{a}{|}^{2}+n_{ab}\cdot 2^{\Upsilon_{b}}|h_{b}{|}^{2}-n_{b,j}\cdot 2^{\Upsilon_{b}}{\left|h_{b}\right|}^{2}\right)\cdot \mathrm{Pr}\left(\Lambda ({\bar{\bar{\Xi }}}_{\chi_{a},z}^{\left(j,1\right)})>0\right)\\ \; & -\mathrm{Pr}\left(-\Lambda \left({\bar{\bar{\Xi }}}_{\chi_{a},z}^{\left(j,1\right)}\right)-\Lambda \left({\tilde{\Xi }}_{\chi_{a},z}^{\left(1\right)}\right)\geq 2^{\Upsilon_{a}}|h_{a}{|}^{2}+n_{ab}\cdot 2^{\Upsilon_{b}}|h_{b}{|}^{2}-n_{b,j}\cdot 2^{\Upsilon_{b}}{\left|h_{b}\right|}^{2}\right)\cdot \mathrm{Pr}\left(\Lambda ({\bar{\bar{\Xi }}}_{\chi_{a},z}^{\left(j,1\right)})<0\right)\\ \geq & 1-\mathrm{Pr}\left(\Lambda \left({\bar{\bar{\Xi }}}_{\chi_{a},z}^{\left(j,1\right)}\right)-\Lambda \left({\tilde{\Xi }}_{\chi_{a},z}^{\left(1\right)}\right)\geq 2^{\Upsilon_{a}}|h_{a}{|}^{2}+n_{ab}\cdot 2^{\Upsilon_{b}}|h_{b}{|}^{2}-n_{b,j}\cdot 2^{\Upsilon_{b}}{\left|h_{b}\right|}^{2}\right)\\ \overset{\left(h\right)}{=} & 1-Q\left(\frac{2^{\Upsilon_{a}}|h_{a}{|}^{2}+n_{ab}\cdot 2^{\Upsilon_{b}}|h_{b}{|}^{2}-n_{b,j}\cdot 2^{\Upsilon_{b}}{\left|h_{b}\right|}^{2}}{\sqrt{2}\sigma_{\Lambda ,1}}\right), \end{array} \end{array}$$

the equal sign (h) is based on $\Lambda \left({\bar{\bar{\Xi }}}_{\chi_{a},z}^{\left(j,1\right)}\right)-\Lambda \left({\tilde{\Xi }}_{\chi_{a},z}^{\left(1\right)}\right)∼N\left(0,2\sigma_{\Lambda ,1}^{2}\right)$. On this basis, we return to (38) and get the final result

(43) $$\begin{array}{c} \begin{array}{cc} \; & \prod_{j=1}^{2^{\Upsilon_{a}}-1}\mathrm{Pr}\left(\left(F_{a}+\Lambda \right)\left({\tilde{\Xi }}_{\chi_{a},z}^{\left(1\right)}\right)+\sum_{z\in F_{2}^{m-\Upsilon_{a}}}\left|F_{b}\left({\tilde{\Xi }}_{\chi_{a},z}^{\left(1\right)}\right)\right|\geq \left|\Lambda ({\bar{\bar{\Xi }}}_{\chi_{a},z}^{\left(j,1\right)})\right|+\sum_{z\in F_{2}^{m-\Upsilon_{a}}}\left|F_{b}\left({\bar{\bar{\Xi }}}_{\chi_{a},z}^{\left(j,1\right)}\right)\right|\right)\\ = & \prod_{j=1}^{2^{\Upsilon_{a}}-1}\left(Q\left(-\frac{2^{\Upsilon_{a}}|h_{a}{|}^{2}+n_{ab}\cdot 2^{\Upsilon_{b}}|h_{b}{|}^{2}-n_{b,j}\cdot 2^{\Upsilon_{b}}{\left|h_{b}\right|}^{2}}{\sqrt{2}\sigma_{\Lambda ,1}}\right)\cdot Q\left(-\frac{2^{\Upsilon_{a}}{\left|h_{a}\right|}^{2}}{\sigma_{\Lambda ,1}}\right)\right). \end{array} \end{array}$$

This equation is consistent with (32) in Lemma 2.

Now we present a special example to provide a further explanation for the result of (43): We suppose that two users have the same submatrix $I_{\chi }$ and different vectors ${\hat{P}}_{1}$; thus, (37) directly becomes

(44) $$\begin{array}{c} \begin{array}{cc} \; & \prod_{j=1}^{2^{\Upsilon_{a}}-1}\mathrm{Pr}\left(\sum_{z\in F_{2}^{m-\Upsilon_{a}}}\left|\left(F_{a}+\Lambda \right)\left({\tilde{\Xi }}_{\chi_{a},z}^{\left(1\right)}\right)\right|>\sum_{z\in F_{2}^{m-\Upsilon_{a}}}\left|\left(F_{b}+\Lambda \right)\left({\bar{\bar{\Xi }}}_{\chi_{a},z}^{\left(j,1\right)}\right)\right|\right)\\ \geq & \prod_{j=1}^{2^{\Upsilon_{a}}-1}\left(\mathrm{Pr}\left(\left(F_{a}+\Lambda \right)\left({\tilde{\Xi }}_{\chi_{a},z}^{\left(1\right)}\right)>\left|F_{b}\left({\bar{\bar{\Xi }}}_{\chi_{a},z}^{\left(j,1\right)}\right)\right|+\left|\Lambda ({\bar{\bar{\Xi }}}_{\chi_{a},z}^{\left(j,1\right)})\right|\right)\cdot \mathrm{Pr}\left(\left(F_{a}+\Lambda \right)\left({\tilde{\Xi }}_{\chi_{a},z}^{\left(1\right)}\right)>0\right)\right)\\ = & {\left(Q\left(-\frac{2^{\Upsilon_{a}}{\left|h_{a}\right|}^{2}}{\sigma_{\Lambda ,1}}\right)\right)}^{2^{\Upsilon_{a}}-1}\cdot \left(\prod_{j=1}^{2^{\Upsilon_{a}}-2}\underset{I}{\underset{︸}{\mathrm{Pr}\left(\left(F_{a}+\Lambda \right)\left({\tilde{\Xi }}_{\chi_{a},z}^{\left(1\right)}\right)>\left|\Lambda ({\bar{\bar{\Xi }}}_{\chi_{a},z}^{\left(j,1\right)})\right|\right)}}\right)\\ & \cdot \underset{II}{\underset{︸}{\mathrm{Pr}\left(\left(F_{a}+\Lambda \right)\left({\tilde{\Xi }}_{\chi_{a},z}^{\left(1\right)}\right)>\left|F_{b}\left({\bar{\bar{\Xi }}}_{\chi_{a},z}^{\left(j,1\right)}\right)\right|+\left|\Lambda ({\bar{\bar{\Xi }}}_{\chi_{a},z}^{\left(j,1\right)})\right|\right)}}\\ = & {\left(Q\left(-\frac{2^{\Upsilon_{a}}{\left|h_{a}\right|}^{2}}{\sigma_{\Lambda ,1}}\right)\right)}^{2^{\Upsilon_{a}}-1}\cdot {\left(Q\left(-\frac{2^{\Upsilon_{a}}{\left|h_{a}\right|}^{2}}{\sqrt{2}\sigma_{\Lambda ,1}}\right)\right)}^{2^{\Upsilon_{a}}-2}\cdot Q\left(-\frac{2^{\Upsilon_{a}}|h_{a}{|}^{2}-2^{\Upsilon_{b}}{\left|h_{b}\right|}^{2}}{\sqrt{2}\sigma_{\Lambda ,1}}\right). \end{array} \end{array}$$

In this case, term *I* refers to interference imposed by the cosets other than set ${\tilde{\Xi }}_{\chi_{a},z}^{\left(1\right)}$ on the $\rho_{a}$-th detected sequence. Since $|{\bar{\bar{\Xi }}}_{\chi_{a},z}^{\left(j,1\right)}\cap {\tilde{\Xi }}_{\chi_{b},z}^{\left(1\right)}|=2^{\Upsilon_{b}}$ for one particular *j*, we conclude that there is only one case in term II. Additionally, owing to $F_{b}\left({\bar{\bar{\Xi }}}_{\chi_{a},z}^{\left(j,1\right)}\right)=0$ for the remaining $\left(2^{\Upsilon_{a}}-2\right)$ possible *j*, it allows $\left(2^{\Upsilon_{a}}-2\right)$ possibilities for the term *I*. The results of (44) satisfy (43) provided that $n_{ab}=0$ and $n_{b,j}=1$ for one *j*, $1\leq j\leq 2^{\Upsilon_{a}}-1$. This equation is also consistent with (32) in Lemma 2.

Based on the above results, after recovering vector ${\hat{P}}_{\rho_{a}}e_{1}$, the $y_{2}$ becomes

(45) $$\begin{array}{c} \begin{array}{cc} y_{2} & =\lambda_{PRM,\rho_{a}}^{\left(1\right)}\cdot y_{1}\\ & =h_{\rho_{a}}C_{{\hat{P}}_{\rho_{a}},B_{\rho_{a}},I_{\chi ,\rho_{a}}}+\lambda_{PRM,\rho_{a}}^{\left(1\right)}\cdot \left(h_{\rho_{b}}C_{{\hat{P}}_{\rho_{b}},B_{\rho_{b}},I_{\chi ,\rho_{b}}}+n\right)\\ & =h_{\rho_{a}}C_{{\hat{P}}_{\rho_{a}},B_{\rho_{a}},I_{\chi ,\rho_{a}}}+\lambda_{PRM,\rho_{a}}^{\left(1\right)}\cdot h_{\rho_{b}}C_{{\hat{P}}_{\rho_{b}},B_{\rho_{b}},I_{\chi ,\rho_{b}}}+\lambda_{PRM,\rho_{a}}^{\left(1\right)}\cdot n, \end{array} \end{array}$$

where $\lambda_{PRM,\rho_{a}}^{\left(1\right)}$ is the first layer projection operator for the user whose detection order is $\rho_{a}$. We can approximate the noise as a Gaussian random variable whose mean is 0 and whose variance is set to be $N_{0}^{\left(\Upsilon_{a}-1\right)}=\frac{|h_{\rho_{b}}{|}^{2}+N_{0}}{2}$. In light of this, the following detection procedure in layer $2\leq r\leq \Upsilon_{a}$ is equivalent to the recovery of the symmetric matrix ${\hat{P}}_{\rho_{a}}^{\Upsilon_{\rho_{a}}-1}$ in a single sequence scenario under the condition that the noise variance is $N_{0}^{\left(\Upsilon_{a}-1\right)}$. Since the $\rho_{a}$-th detected sequence can be successfully recovered if and only if both ${\hat{P}}_{\rho_{a}}e_{1}$ and ${\hat{P}}_{\rho_{a}}^{\Upsilon_{\rho_{a}}-1}$ are correct, the successful detection probability of event “the $\rho_{k}$-th recovered sequence has been successfully detected” is

(46) $$\begin{array}{c} \begin{array}{cc} \; & \mathrm{Pr}\left(B_{\rho_{a}}|\underline{h}\right)=\mathrm{Pr}\left(P_{\rho_{a}}^{\left(1\right)}|\underline{h}\right)\cdot \overset{ˇ}{P}\left(\Upsilon_{\rho_{a}}-1,N_{0}^{\left(\Upsilon_{a}-1\right)}|\underline{h}\right). \end{array} \end{array}$$

This result is consistent with (31) in Lemma 2.

Next, we assume that the sequence $C_{{\hat{P}}_{\rho_{a}},B_{\rho_{a}},I_{\chi ,\rho_{a}}}$ is ideally eliminated from the signal y, the receiver proceeds to recover $C_{{\hat{P}}_{\rho_{b}},B_{\rho_{b}},I_{\chi ,\rho_{b}}}$, and we denote its probability of successful detection as $\mathrm{Pr}\left(B_{\rho_{b}}|B_{\rho_{a}},\underline{h}\right)=\overset{ˇ}{P}\left(\Upsilon_{\rho_{b}},N_{0}|h_{\rho_{b}}\right)$.

As a next step, we will calculate the average probability of two sequences *a* and *b* being detected successfully. In the following two scenarios, the sequences *a* can be recovered:

- In the first instance, sequence *a* is detected successfully first, which has a chance of $\mathrm{Pr}\left(B_{a}|\underline{h}\right)$.
- In the second instance, sequence *b* is detected in priority with the probability of $\mathrm{Pr}\left(B_{b}|\underline{h}\right)$, followed by sequence *a* being detected from the subtracted signal with the probability $\mathrm{Pr}\left(B_{a}|B_{b},\underline{h}\right)$.

As a result, the probability that sequence *a* will be successfully detected equals

(47) $$\begin{array}{c} \begin{array}{c} \overset{ˇ}{\overset{ˇ}{P}}\left(K_{a},m,N_{0}|\underline{h}\right)=\mathrm{Pr}\left(B_{1}|\underline{h}\right)+\mathrm{Pr}\left(B_{2}|\underline{h}\right)\cdot \mathrm{Pr}\left(B_{1}|B_{2},\underline{h}\right). \end{array} \end{array}$$

The equation is consistent with (34) in Theorem 2. Finally, the average probability of successful detection equals

(48) $$\begin{array}{c} \begin{array}{c} {\overset{ˇ}{\overset{ˇ}{P}}}_{\mathrm{ave}}\left(K_{a},m,N_{0}|\underline{h}\right)=\frac{1}{2}\int_{\underline{h}}\left\{\prod_{\rho =1}^{2}f_{\mathrm{CN}}\left(h_{\rho };0.1\right)\cdot \left({\overset{ˇ}{\overset{ˇ}{P}}}_{1}\left(K_{a},m,N_{0}|\underline{h}\right)+{\overset{ˇ}{\overset{ˇ}{P}}}_{2}\left(K_{a},m,N_{0}|\underline{h}\right)\right)\right\}. \end{array} \end{array}$$

The equation is consistent with (35) in Theorem 2. □

## 5. Simulation Results

In this section, we first examine the effectiveness of the proposed algorithms by evaluating the numerical simulation of the successful detection probability in Section 5.1. Additionally, in Section 5.2, a slot-controlled CCS scheme with the proposed iterative list projection algorithm in place of the origin inner decoder has been measured against several benchmarks.

### 5.1. Performances of the Algorithms 1 and 2

In this section, we will examine the detection capability of Algorithms 1 and 2, and our proposed scheme’s performance is determined by computing the successful access probability. In the sequel, the received signal-to-noise ratio (SNR) at the AP is given by

(49) $$\begin{array}{c} \begin{array}{c} \mathrm{SNR}\left(\mathrm{dB}\right)=E\left[10\mathrm{lg}\frac{∥y^{\left(t\right)}-e^{\left(t\right)}{∥}^{2}}{∥e^{\left(t\right)}{∥}^{2}}\right]. \end{array} \end{array}$$

The successful detection probability is the mean proportion of determined active users to the system’s overall number of active users.

**Figure 6:**
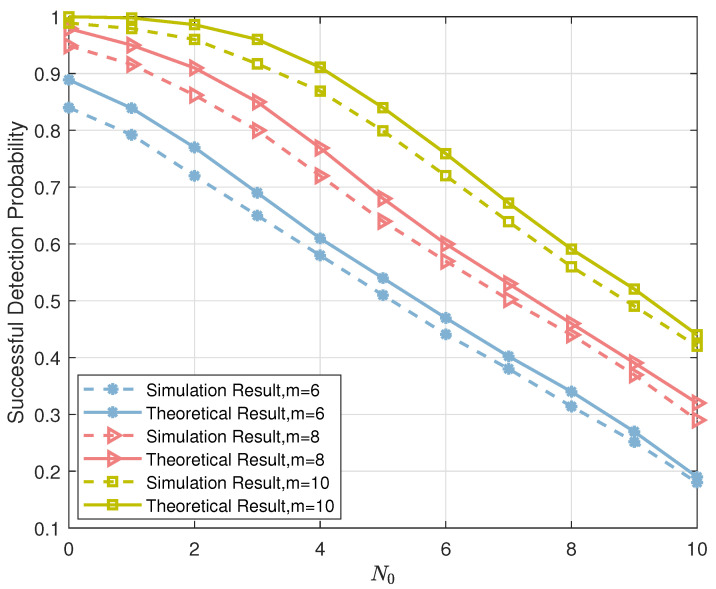
The comparison of numerical simulation results and theoretical analysis results of list PRM projection algorithm.

As for the performance of Algorithm 1: Figure 6 compares the numerical simulation results of the list PRM projection algorithm (Algorithm 1) with the theoretical analysis results based on (13) for different settings of *m*. We fix Υ=m, H=1, and the transmit power of an active user is normalized to one. This figure illustrates that, although we employed Gaussian approximation during the theoretical analysis process, the numerical simulation results and the theoretical analysis are closely matched, and definitely, the difference between the two simulations is the result of the Gaussian approximations. Thus, this result confirms the correctness of the theoretical analysis in Lemma 1. In addition, comparing the successful detection probability for different *m*, we find that the performance of the list PRM projection algorithm improves as the sequence length increases, further validating the conclusion in Section 4.1.

**Figure 7:**
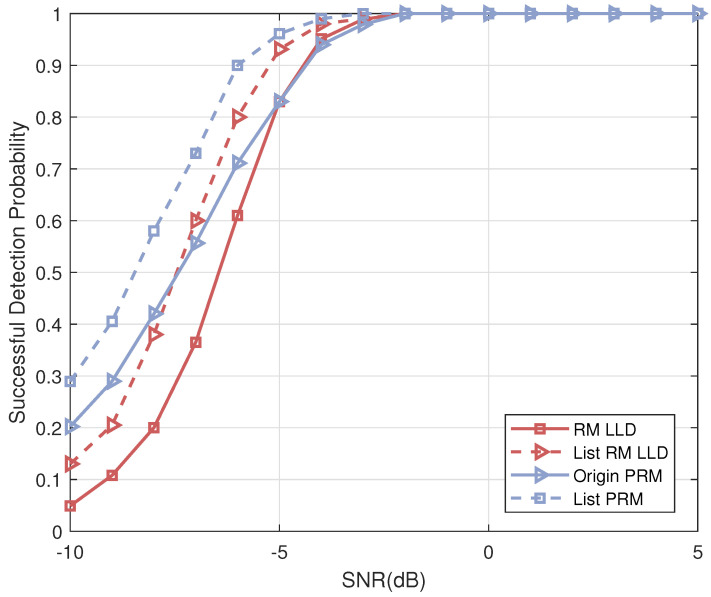
The successful detection probability versus SNR for different schemes, m=8.

Next, we will compare the performance of the list PRM projection algorithm (Algorithm 1) with the existing decoding compressed sensing algorithm for RM sequences as the common codebook for all active users in a random access system. The numerical simulation results are shown in Figure 7, where “Origin PRM” and “List PRM” represent the origin projection detection algorithm in [37] and its list version (Algorithm 1), respectively. “RM LLD” represents the RM sequence detection algorithm proposed in Reference [33], which is based on the shift-and-multiply operation on the received signal, and “List RM LLD” represents its list version detection. For all algorithms, we set the sequence length to $N=2^{8}$ and the list size to H=8. As can be seen from Figure 7, the successful detection probabilities of all algorithms are close to one when the signal-to-noise ratio (SNR) is larger than −2 dB. However, the “RM LLD” algorithm suffers from severe performance degradation under low SNR conditions compared to the “Origin PRM”, which shows strong robustness. In addition, due to the enhanced list detection, the “List RM LLD” and the “List PRM” provide a more outstanding performance gain under lower SNR when compared with no candidate-saving algorithms.

**Figure 8:**
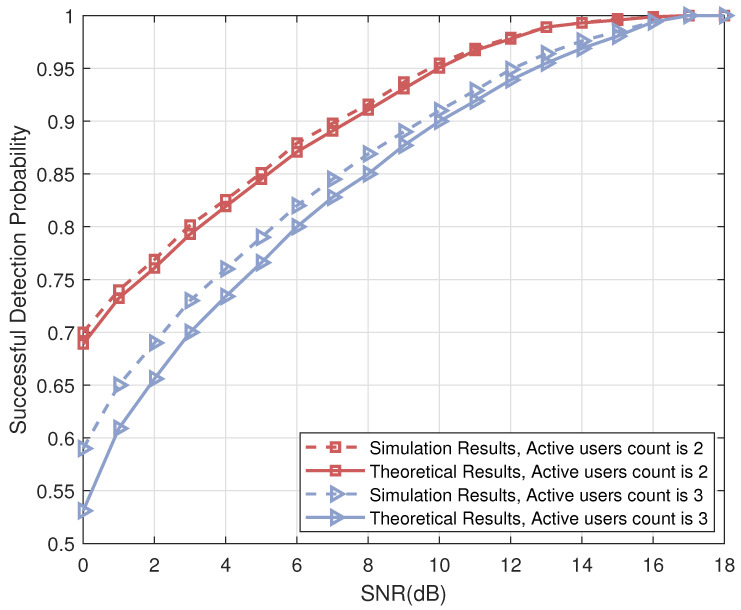
The successful detection probability of Algorithm 1 versus SNR.

As for the performance of Algorithm 2: in Figure 8, we compare the numerical simulation results of the iterative list PRM projection detection (Algorithm 2) with the theoretical analysis results derived from Lemma 2. We set the number of iterations in the numerical simulation 1. From Figure 8, the theoretical analysis results fit the numerical simulation results well, especially under high SNR conditions, thus verifying the correctness of Theorem 2.

In addition, both simulation and theoretical results show that the detection capability of the algorithm decreases as the number of active users in the system increases.

### 5.2. Iterative List PRM Projection Algorithm-Based Slot-Controlled URA

**Figure 9:**
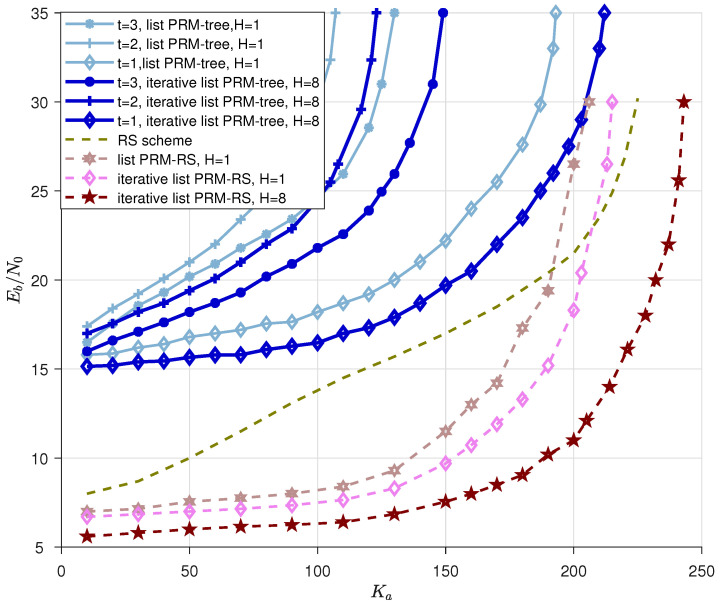
The simulations of energy efficiency are depicted. The simulations are listed as follows: list PRM-tree scheme for t=1,⋯,3 from [37], iterative list PRM-tree scheme for t=1,⋯,3, Reed-Solomon scheme from [27], list PRM-RS scheme without iteration form [37], and iterative list PRM-RS schemes for H=1 and H=8, respectively.

In this section, we combine an inner code employing an iterative list PRM projection detector as the decoder with two types of practicable outer codes (*t*-tree and Reed–Solomon codes) to form a slot-pattern control-based CCS system (as illustrated in Section 2). We demonstrate the positive effects of the proposed algorithms on the overall system via simulation results. We consider a communication system in which each user transmits B=100 bits by employing a $H_{N}$ length *G*-ary outer-code using $T=2^{15}$ channel uses. The $H_{N}$ positions are carefully selected within $N_{T}$ chunks. There are *G* codewords in the inner codebook, each of which has a length of $N=T/N_{T}$. We employ the PRM sequence under one rank Υ rather than all ranks in this section, and Algorithm 2 is used to decode the inner codes for multi-sequence scenarios. The energy efficiency of different schemes is plotted as follows: energy efficiency is the minimum $E_{b}/N_{0}$ over the possible scheme-specific parameters in the URA scheme, $P_{e}<0.1$ is the probability of error constraint for each user, and $P_{f}<0.001$ is the FAR (false-alarm rate) constraint. Numerical results are presented in Figure 9.

This section first compares the numerical simulations of the enhanced slot-controlled URA system where the outer-code is a *t*-tree code with some benchmarks. We cite benchmarks as follows: We denote a “list PRM-tree” scheme that concatenates the inner-code with an OMP decoder to an outer decoder that can correct up to *t* errors for various setups, i.e., the benchmarks called “t=3, list PRM-tree, H=1”, “t=2, list PRM-tree, H=1” and “t=1, list PRM-tree, H=1” are plotted in light blue in Figure 9. These benchmarks are drawn from Reference [27] and named “PRM-RS” in Reference [27]. We employ blue lines to illustrate the simulations for the proposed “t=3, iterative list PRM-tree, H=8”, “t=2, iterative list, H=8” and “t=1, iterative list PRM-tree, H=8” schemes. Specifically, for these proposed “iterative list PRM-tree, H=8” schemes, the PRM sequences are employed as the inner codewords, in which the former $x_{p}$-bit message is filled with the selected SPC, and the remaining *J*-bit is used to transport the user’s messages. The enhanced Iterative list PRM projection algorithm is adopted in order to jointly recover the list of messages and the channel estimations of all sequences within one slot. Moreover, the decoder capable of correcting *t* errors continues to make up the outer code. A greedy information bit allocation method is applied to all list PRM-tree schemes, where each subsequent slot is assigned the maximum amount of information bits while maintaining the $E[|V_{l}\left|\right]\leq 2^{25}$ constraint and finding the minimum $E_{b}/N_{0}$. The energy efficiency is the minimum power needed to serve $K_{a}$ users with the per-user probability of error less than 0.1, we depict the curves in the blue line in Figure 9 under the optimal setups. The optimal setups of $H_{N}$, *N*, $N_{T}$, *G*, *J* and $x_{p}$ are shown in Table 2. From Table 2, we find that the outer-code length $H_{N}=32$ is used for t=1 and $H_{N}=64$ for t=2,3, which is due to the fact that the latter case performs relatively poorly since it has a greater number of simultaneous appearances, which leads to inner-code failure at $K_{a}=185$ for t=1, $K_{a}=110$ and $K_{a}=130$ for t=2 and t=3, respectively, under “list PRM-tree, H=1” schemes; $K_{a}=220$ for t=1, $K_{a}=125$ and $K_{a}=150$ for t=2 and t=3, respectively, when conducting “iterative list PRM-tree, H=8” schemes.

Next, we will compare the numerical simulations of the enhanced slot-controlled URA system where the outer-code is a Reed–Solomon code with some benchmarks. We cite benchmarks as follows: We denote the CCS scheme that concatenates the inner codes whose distribution is i.i.d. uniform on the power shell to an outer Reed–Solomon code as the “RS scheme” (it is named ”Reed–Solomon scheme” in Reference [27]), and we use it as the first benchmark. Furthermore, the scheme called “list PRM-RS, H=1” is directly drawn from Reference [37] as the second benchmark (it is named “PRM-RS” in Reference [37]), and this slot-controlled URA scheme ”list PRM-RS, H=1” that is described in the system model (Section 2) does not employ the enhanced detection algorithms. Additionally, the “iterative list PRM-RS, H=1” scheme is the proposed scheme whose multi-user inner-code detector is substituted by Algorithm 2 without saving candidates while calling Algorithm 1 (since H=1), whereas the proposed “iterative list PRM-RS, H=8” scheme keeps H=8 candidates during each layer while conducting Algorithm 1. The optimal setups of “list PRM-RS, H=1”, “iterative list PRM-RS, H=1” and “iterative list PRM-RS, H=8” are shown in Table 3, and the energy efficiency outcomes are illustrated in Figure 9.

From the simulations depicted in Figure 9, the following observations can be drawn:

1. As for all “List PRM-tree” schemes, since the outer-code length $H_{N}=32$ is suitable for scheme t=1 and $H_{N}=64$ for t=2,3 cases, the former case (t=1 case) can accommodate more active users than t=2,3 owing to a smaller number of simultaneous appearances in each slot (the outer code length affects the probability of appearance over slots. See more setup details in Table 2). Additionally, a higher *t* for the outer code increases performance when the inner code has the same length. Therefore, t=3 is superior to t=2. Furthermore, when comparing the curves of “list PRM-tree” and “iterative list PRM-tree”, we conclude that the enhanced inner code with the proposed iterative list projection algorithm can help the entire system lower the energy-per-bit requirement to meet a target error probability as well as boost the ability to accommodate more users’ transmission.
2. Figure 9 illustrates how the “iterative list PRM-RS, H=1” curve outperforms the “list PRM-RS, H=1” curve regarding energy consumption when the number of active users is fixed. This observation is an unambiguous demonstration of the benefit of Algorithm 2, which carries out iterative detection.
3. When compared with different schemes of “iterative list PRM-RS, H=1” and “iterative list PRM-RS, H=8”, we demonstrate that the performance of the detection can be refined by raising the count of candidates in the first few layers, thereby confirming the significance of Algorithm 1, which guarantees the first few layers’ reliable recoveries.

**Figure 10:**
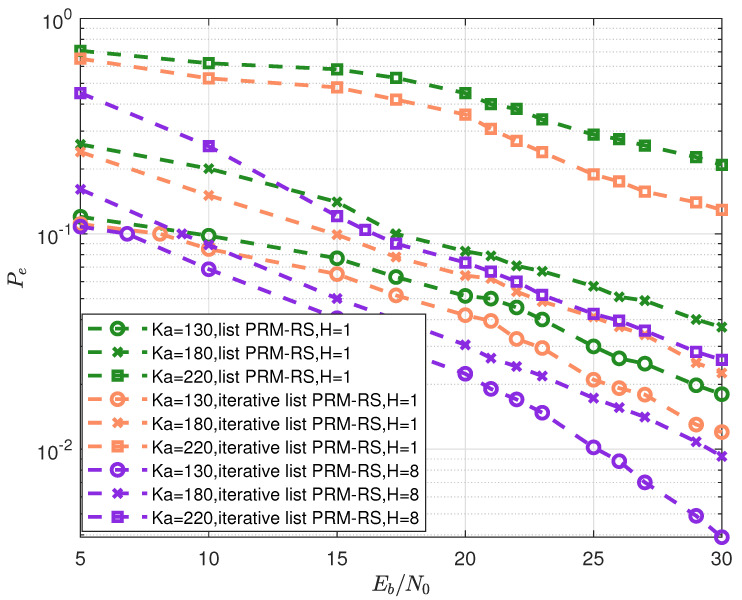
Probability of error $P_{e}$ vs. $E_{b}/N_{0}$ for $K_{a}=130,180$ and 220 under three schemes, i.e., “list PRM-RS, H=1”, “iterative list PRM-RS, H=1” and “iterative list PRM-RS, H=8”.

The per-user error probability $P_{e}$ as a function of $E_{b}/N_{0}$ for $K_{a}=130,180$ and 200 for the “list PRM-RS, H=1”, “iterative list PRM-RS, H=1” and “iterative list PRM-RS, H=8” schemes are obtained (in Figure 10). The optimal system parameters are depicted in Table 3. It can be seen from Figure 10 that:

1. For “list PRM-RS, H=1” and “iterative list PRM-RS, H=1” schemes, the minimum $P_{e}$ exceeds 0.1 when the user count reaches 220. Besides, the minimum $P_{e}$ for “iterative list PRM-RS, H=8” schemes below 0.1 when $E_{b}/N_{0}$ is beyond 16dB.
2. The simulation curves in Figure 10 are consistent with the performances in Figure 9, i.e., for “list PRM-RS, H=1” and “iterative list PRM-RS H=1” schemes, when the number of users exceeds 205 and 213, respectively, the number of simultaneous transmissions exceeds the identifiable maximum.
  For “iterative list PRM-RS, H=8” schemes, when the number of users exceeds 243, the number of simultaneous transmissions exceeds the identifiable maximum.

## 6. Conclusions

In this paper, we address a packetized and slotted transmission CCS framework which concatenates an inner PRM code to an outer error correction code. First, we improve the decoder of the inner-code: we propose an enhanced algorithm that makes use of multiple candidates so as to remedy the venture of spreading errors. On this basis, an iterative list PRM projection algorithm is proposed for the multi-sequence scenario. We then deduce the theoretical probabilities of the list projection and iterative list projection detection methods. Via numerical simulations, we indicate the theoretical and the simulation results agreed. Finally, we implement the iterative list PRM projection algorithm in two practical error-correction codes. From the simulation results, we conclude that the packetized URA with the proposed iterative list projection detection works better than benchmarks in terms of the number of active users it can support in each slot and the amount of energy needed per bit to meet an expected error probability.

Additional interesting research avenues include: (i) the PRM-based system can be expanded to support multiple antennas; (ii) the decomposition of the Clifford matrix $G_{F}$ associated with the patterned RM code can be considered as a transmitter’s information reconstruction, and the iterative approach is generalized at the receiver.

## Figures and Tables

**Table 1:** Uncoordinated/unsourced grant-free vs. coordinated/sourced grant-free (based on compressed sensing technique).

Schemes	Application Scenarios	Typical Access Performance	Advantages and Disadvantages
Uncoordinated/unsourced grant-free [8]	Providing complete data transfer for active users in a large-scale random access environment	It is possible to achieve a BER of 0.05 using 30,000 channels, a 3 dB SNR, and 200 active users	Advantages: can support a large number of total users, does not need user identity recovery process, and is suitable for low latency transmission scenarios Disadvantages: no pilot sequence, hard to estimate the channel estimations, and high complexity of decoders
Coordinated/sourced grant-Free based on Compressed sensing technique [9,10,11,12]	Providing active user identity detection and channel estimation in a large-scale random access environment	Using a pilot sequence of length-800, an SNR of 3 dB, and a setup of 40,000 users, it is possible to achieve a BER of 0.0125	Advantage: can jointly recover users’ ID and their channel estimations Disadvantages: the overhead of the pilot sequence is large, and the total number of supported users is constrained by the length of the pilot sequence

**Table 2:** URA system parameters for “PRM-tree” scheme.

Parameter Description	Specific Value
PRM sequence length, $N=2^{m}$	$2^{7}$
The length of RS codes, $H_{N}$	$2^{5}\left(t=1\right)/2^{6}\left(t=2,3\right)$
The number of slots, $N_{T}$	$2^{8}$
The number of complex channel uses, *T*	$2^{15}$
The length of slot-occupation control, $x_{p}$	9(t=1)/7(t=2,3)
*G*-ary	19(t=1)/15(t=2,3)
*J*-ary	10(t=1)/8(t=2,3)
The capacity of PRM codebook, $|\Gamma_{\Upsilon }|$	286720(t=1)/21504(t=2,3)
The code rate	0.3125(t=1)/0.1953(t=2,3)

**Table 3:** URA system parameters for “RS” scheme.

Parameter Description	Specific Value
PRM sequence length, $N=2^{m}$	$2^{7}$
The length of RS codes, $H_{N}$	$2^{5}$
The number of slots, $N_{T}$	$2^{8}$
The number of complex channel uses, *T*	$2^{15}$
The length of slot-occupation control, $x_{p}$	9
*G*-ary	15
*J*-ary	6
The capacity of PRM codebook, $|\Gamma_{\Upsilon }|$	21504
The code rate of RS	0.5208

## Data Availability

Not applicable.

## References

[1] Saad W., Bennis M., Chen M. (2019). A vision of 6G wireless systems: Applications, trends, technologies, and open research problems. IEEE Netw..

[2] Guo F., Yu F.R., Zhang H., Li X., Ji H., Leung V.C. (2021). Enabling massive IoT toward 6G: A comprehensive survey. IEEE Internet Things J..

[3] Pan C., Mehrpouyan H., Liu Y., Elkashlan M., Arumugam N. (2018). Joint pilot allocation and robust transmission design for ultra-dense user-centric TDD C-RAN with imperfect CSI. IEEE Wirel. Commun..

[4] Chettri L., Bera R. (2020). A comprehensive survey on Internet of Things (IoT) toward 5G wireless systems. IEEE Internet Things J..

[5] Masoudi M., Azari A., Yavuz E.A., Cavdar C. Grant-free radio access IoT networks: Scalability analysis in coexistence scenarios. Proceedings of the IEEE International Conference on Communications (ICC).

[6] Shahab M.B., Abbas R., Shirvanimoghaddam M., Johnson S.J. (2020). Grant-free non-orthogonal multiple access for IoT: A survey. IEEE Commun. Surv. Tut..

[7] Chen X., Chen T.Y., Guo D. (2017). Capacity of Gaussian many-access channels. IEEE Trans. Inf. Theory.

[8] Polyanskiy Y. A perspective on massive random-access. Proceedings of the IEEE International Symposium on Information Theory (ISIT).

[9] Chen Z., Sohrabi F., Yu W. (2018). Sparse activity detection for massive connectivity. IEEE Trans. Signal Process..

[10] Liu L., Yu W. (2018). Massive connectivity with massive MIMO—Part I: Device activity detection and channel estimation. IEEE Trans. Signal Process..

[11] Senel K., Larsson E.G. (2018). Grant-free massive MTC-enabled massive MIMO: A compressive sensing approach. IEEE Trans. Commun..

[12] Yang L., Fan P., Li L., Ding Z., Hao L. (2021). Cross validation aided approximated message passing algorithm for user identification in mMTC. IEEE Commun. Lett..

[13] Zadik I., Polyanskiy Y., Thrampoulidis C. Improved bounds on Gaussian MAC and sparse regression via Gaussian inequalities. Proceedings of the 2019 IEEE International Symposium on Information Theory (ISIT).

[14] Ngo K.H., Lancho A., Durisi G. (2023). Unsourced multiple access with random user activity. IEEE Trans. Inf. Theory.

[15] Ordentlich O., Polyanskiy Y. Low complexity schemes for the random access Gaussian channel. Proceedings of the 2017 IEEE International Symposium on Information Theory (ISIT).

[16] Marshakov E., Balitskiy G., Andreev K., Frolov A. A polar code based unsourced random access for the Gaussian MAC. Proceedings of the 2019 IEEE 90th Vehicular Technology Conference (VTC2019-Fall).

[17] Pradhan A.K., Amalladinne V.K., Vem A., Narayanan K.R., Chamberland J.F. (2022). Sparse IDMA: A Joint Graph-Based Coding Scheme for Unsourced Random Access. IEEE Trans. Commun..

[18] Ahmadi M.J., Duman T.M. (2021). Random spreading for unsourced MAC with power diversity. IEEE Commun. Lett..

[19] Pradhan A.K., Amalladinne V.K., Narayanan K.R., Chamberland J.F. LDPC Codes with Soft Interference Cancellation for Uncoordinated Unsourced Multiple Access. Proceedings of the IEEE International Conference on Communications (ICC).

[20] Amalladinne V.K., Chamberland J.F., Narayanan K.R. (2020). A Coded Compressed Sensing Scheme for Unsourced Multiple Access. IEEE Trans. Inf. Theory.

[21] Amalladinne V.K., Vem A., Soma D.K., Narayanan K.R., Chamberland J.F. A coupled compressive sensing scheme for unsourced multiple access. Proceedings of the 2018 IEEE International Conference on Acoustics, Speech and Signal Processing (ICASSP).

[22] Lancho A., Fengler A., Polyanskiy Y. Finite-blocklength results for the A-channel: Applications to unsourced random access and group testing. Proceedings of the 2022 58th Annual Allerton Conference on Communication, Control, and Computing (Allerton).

[23] Amalladinne V.K., Pradhan A.K., Rush C., Chamberland J.F., Narayanan K.R. (2022). Unsourced random access with coded compressed sensing: Integrating AMP and belief propagation. IEEE Trans. Inf. Theory.

[24] Andreev K., Rybin P., Frolov A. Reed-Solomon coded compressed sensing for the unsourced random access. Proceedings of the 2021 17th International Symposium on Wireless Communication Systems (ISWCS).

[25] Che J., Zhang Z., Yang Z., Chen X., Zhong C., Ng D.W.K. (2021). Unsourced random massive access with beam-space tree decoding. IEEE J. Sel. Areas Commun..

[26] Fengler A., Haghighatshoar S., Jung P., Caire G. (2021). Non-Bayesian Activity Detection, Large-Scale Fading Coefficient Estimation, and Unsourced Random Access With a Massive MIMO Receiver. IEEE Trans. Inf. Theory.

[27] Andreev K., Rybin P., Frolov A. (2022). Coded Compressed Sensing with List Recoverable Codes for the Unsourced Random Access. IEEE Trans. Commun..

[28] Liang Z., Zheng J., Ni J. (2021). Index modulation–aided mixed massive random access. Front. Commun. Networks.

[29] Fengler A., Jung P., Caire G. (2021). SPARCs for unsourced random access. IEEE Trans. Inf. Theory.

[30] Ebert J.R., Amalladinne V.K., Rini S., Chamberland J.F., Narayanan K.R. (2022). Coded demixing for unsourced random access. IEEE Trans. Signal Process..

[31] Zhang L., Luo J., Guo D. (2013). Neighbor discovery for wireless networks via compressed sensing. Perform. Eval..

[32] Zhang H., Li R., Wang J., Chen Y., Zhang Z. Reed-Muller sequences for 5G grant-free massive access. Proceedings of the GLOBECOM 2017 IEEE Global Communications Conference.

[33] Wang J., Zhang Z., Hanzo L. (2019). Joint active user detection and channel estimation in massive access systems exploiting Reed–Muller sequences. IEEE J. Sel. Top. Signal Process..

[34] Wang J., Zhang Z., Hanzo L. (2020). Incremental massive random access exploiting the nested Reed-Muller sequences. IEEE Trans. Wirel. Commun..

[35] Calderbank R., Thompson A. (2020). CHIRRUP: A practical algorithm for unsourced multiple access. Inform. Inference J. IMA.

[36] Wang J., Zhang Z., Chen X., Zhong C., Hanzo L. (2020). Unsourced massive random access scheme exploiting reed-muller sequences. IEEE Trans. Commun..

[37] Xie W., Zhang H. (2023). Patterned Reed–Muller Sequences with Outer A-Channel Codes and Projective Decoding for Slot-Controlled Unsourced Random Access. Sensors.

[38] Pllaha T., Tirkkonen O., Calderbank R. (2022). Binary subspace chirps. IEEE Trans. Inf. Theory.

[39] Pllaha T., Tirkkonen O., Calderbank R. Reconstruction of multi-user binary subspace chirps. Proceedings of the 2020 IEEE International Symposium on Information Theory (ISIT).

[40] Tirkkonen O., Calderbank R. Codebooks of complex lines based on binary subspace chirps. Proceedings of the 2019 IEEE Information Theory Workshop (ITW).

